# Comparative transcriptome profiling and weighted gene co-expression network analysis to identify core genes in maize (*Zea mays* L.) silks infected by multiple fungi

**DOI:** 10.3389/fpls.2022.985396

**Published:** 2022-10-27

**Authors:** Amrendra Kumar, Kanak Raj Kanak, Annamalai Arunachalam, Regina Sharmila Dass, P. T. V. Lakshmi

**Affiliations:** ^1^ Phytomatics Lab, Department of Bioinformatics, School of Life Sciences, Pondicherry University, Pondicherry, India; ^2^ Fungal Genetics and Mycotoxicology Laboratory, Department of Microbiology, School of Life Sciences, Pondicherry University, Puducherry, India; ^3^ Postgraduate and Research Department of Botany, Arignar Anna Government Arts College, Villupuram, Tamil Nadu, India

**Keywords:** transcriptome analysis, WGCNA, CAZymes, R-gene, transcription factor, fungal, maize silks

## Abstract

Maize (*Zea mays* L.) is the third most popular *Poaceae* crop after wheat and rice and used in feed and pharmaceutical sectors. The maize silk contains bioactive components explored by traditional Chinese herbal medicine for various pharmacological activities. However, *Fusarium graminearum*, *Fusarium verticillioides*, *Trichoderma atroviride*, and *Ustilago maydis* can infect the maize, produce mycotoxins, hamper the quantity and quality of silk production, and further harm the primary consumer’s health. However, the defense mechanism is not fully understood in multiple fungal infections in the silk of *Z. mays*. In this study, we applied bioinformatics approaches to use the publicly available transcriptome data of *Z. mays* silk affected by multiple fungal flora to identify core genes involved in combatting disease response. Differentially expressed genes (DEGs) were identified among intra- and inter-transcriptome data sets of control *versus* infected *Z. mays* silks. Upon further comparison between up- and downregulated genes within the control of datasets, 4,519 upregulated and 5,125 downregulated genes were found. The DEGs have been compared with genes in the modules of weighted gene co-expression network analysis to relevant specific traits towards identifying core genes. The expression pattern of transcription factors, carbohydrate-active enzymes (CAZyme), and resistance genes was analyzed. The present investigation is supportive of our findings that the gene ontology, immunity stimulus, and resistance genes are upregulated, but physical and metabolic processes such as cell wall organizations and pectin synthesis were downregulated respectively. Our results are indicative that terpene synthase TPS6 and TPS11 are involved in the defense mechanism against fungal infections in maize silk.

## Introduction

Maize (*Zea mays* L.), also called the “queen of cereals”, ranks third in the world after wheat and rice production. About 5.5% of maize (corn) is used as human food from all energy sources of food (51%), which comes from rice (20%), wheat (20%), and other cereals or grains (6%). It is also one of the most widely grown grain crop and is being cultivated in more than 166 countries. The United States produces most of the maize (30%), followed by China (23%), Brazil (9%), Argentina (5%), and India (2%) (Crop et al., 2021). Maize is primarily grown for food and feed intent for human and animal nutrition. In addition, maize has found extensive applications in beauty and drug industries, too. All plant parts in *Z. mays* can be used to generate revenue. The silk from *Z. mays* has been used to treat different illnesses as it is being applied in the Indian system of medicine and Chinese traditional medicine (Zhao et al., 2012). In fact, in India, between 2,500 BCE and 500 BCE, the ayurvedic concept saw *Z. mays* as an essential herb, especially the silk part (stigma maydis), for healing and controlling many diseases (Pandey et al., 2013). This traditional knowledge of the significance of the use of maize silk was eventually lost with the advent of allopathy in the 18th century. Recent research has shown that maize silk exhibits powerful health-promoting effects. This is also true because the silk contains bioactive compounds such as flavonoids, proteins, carbohydrates, vitamins, steroids, tannins, alkaloids, mineral salts, and polysaccharides (Zhao et al., 2012; Guo et al., 2017). These compounds may help protect against cancer, hypertension, diabetes, hepatic, cardiovascular, and other age-related diseases. Researchers are exploring ways to lower body weight and blood glucose levels, increase serum insulin secretion, improve glucose intolerance in type 2 diabetic mice, and control hyperglycemia (Mada et al., 2020).

Rahman and Wan Rosli (2014) opined that maize silk provided an ideal environment for fungal propagules as a nutrient-rich, soft, and moisture-laden tissue within the husks. The corn silk serves as an ideal place for fungal propagules to reside and multiply within the cob environment. The fungal spores adhere and germinate into hyphal structures, which spreads into the maize silk, infects the ovules, and creates an imbalance of the hormones (Li et al., 2018). The parenchymatous cells of the maize silk serves as a suitable place for hyphae to grow especially for fungi like *Aspergillus*, *Fusarium*, *Penicillium*, and *Ustilago* species which cause diseases like ear rot, corn smut, and brown spot (Miller et al., 2007) and an economic loss of 5–42% yield per year (Thompson and Raizada, 2018). The silk of *Z. mays* is also said to have an effect against *Trichoderma* species (Gong et al., 2014; Contreras-Cornejo et al., 2016).

A study in 2019 reported that maize silk has the genes and transcription factors that code for the callose of the papillae, which prevent fungi from growing (Shi et al., 2019) within the cobs. *Fusarium* species like *Fusarium graminearum* (Fg) and *Fusarium verticillioides* (Fv) usually infect the outer layer of the maize silk in *Z. mays* in order to draw nourishment for hyphal growth. On the other hand, infection caused by *Ustilago maydis* was found to affect the entire length of maize silk. These fungi produce mycotoxins, carcinogenic substances that cause esophageal and liver inflammation in humans (Marín et al., 2004). It is therefore important to understand the mechanism of the plant–fungal interaction in the infection process. Transcriptome studies have been extensively used to study specific genes expressed during the infection process. Hence, multiple fungal systems from the families *Nectriaceae* (*F. verticillioides*—Fv and *F. graminearum*—Fg), *Hypocreaceae* (*Trichoderma atroviride*—Ta), and *Ustilaginaceae* (*U. maydis*—Um) were used to study the expression pattern in *Z. mays* silk. Agostini et al. (2019) chose the datasets from the experimental studies on the combination of these fungi to examine and figure out the essential genes involved in many molecular and biological processes. Furthermore, we looked at the co-expression in different networks using weighted gene co-expression network analysis (WGCNA) to build the networks based on the pairwise co-expression between gene expression levels. Since WGCNA builds a scale-free network based on similarities in gene expression profiles that may be linked to the phenotypes of interest, this method was used to find groups of genes that work well together (Bakhtiarizadeh et al., 2018; Abbassi-Daloii et al., 2020; Xu et al., 2021). The goal of the comparative study based on statistical estimates like the DEG and WGCNA modules was to investigate and study more about how different fungi infect *Z. mays* silk. *F. graminearum*, *F. verticillioides*, and *U. maydis* are all partially biotrophic parasites that can also eat dead organisms (Incremona et al., 2014; Pei et al., 2019; Pandian et al., 2020). *F. verticillioides* is an endophyte which competes with the fungal pathogen *F. graminearum* and is antagonistic to *U. maydis* (Lee et al., 2009; Rodriguez Estrada et al., 2012). This study evaluates how fungal stress resistance and yield can improve maize silk through molecular breeding and biotechnology.

## Material and methods

### RNA-Seq data collection, pre-processing, and alignment

Two transcriptome datasets of fungus-infected silk of *Z. mays* were obtained from the National Centre for Biotechnology Information-Sequence Read Archive (NCBI SRA) database. Each dataset consisted of samples (18) infected by different fungi belonging to the families of *Hypocreaceae*, *Nectriaceae*, and *Ustilaginaceae*, with BioProject accession numbers PRJNA13048 (A) (https://www.ncbi.nlm.nih.gov/bioproject/PRJEB13048) and PRJNA382306 (B) (https://www.ncbi.nlm.nih.gov/bioproject/PRJNA362306). Each data set had three biological replicates comprising of silk samples affected with two fungi, *F. graminearum* and *U. maydis* in A, while B was infected with *F. verticillioides* and *T. atroviride* along with the control in each dataset (Agostini et al., 2019) (**Supplementary Table S1A**). The quality of both datasets was computed using the FASTQC tool (http://www.bioinformatics.babraham.ac.uk/projects/fastqc/), and the raw read sequences of both datasets were mapped to the latest reference sequence of *Z. mays* B73 (V5.0) (http://ftp.ebi.ac.uk/ensemblgenomes/pub/release-51/plants/fasta/zea_mays) using the HISAT2 tool (Kim et al., 2015). The read count of each gene mapped to the reference genome was calculated using the FeatureCount tool (Liao et al., 2014).

### Differential gene expression analysis and identification of common genes

Pairwise differential gene expression analysis was performed using control A and B datasets based on the experimental design (**Supplementary Table S1B**). DESeq2 of Bioconductor R package (Love et al., 2014) was used to perform differential expression calculation, and significantly differentially expressed genes (DEGs) were identified by applying the cutoff value of log_2_ fold change of ≥|1.5| with *P*-value cutoff <0.05. The expected significant common DEGs between control A and B datasets were filtered out for further analysis.

### WGCNA analysis

Weighted gene co-expression network analysis (WGCNA) (Langfelder and Horvath, 2008) of the R package was performed on the complete read count matrix of common DEGs identified (12,447 genes across 18 samples). The matrix between each pair of genes across all the samples was calculated using Pearson’s correlation. It generated an adjacency matrix by default soft power and computed the topological overlap matrix (TOM) along with the corresponding dissimilarity (1-TOM) values. Gene modules were detected using the dynamic cutting algorithm with a minimum module size of 30 and a default cutoff height (0.99), and the gene modules were arranged in the dendrogram depending on their shape (Langfeldera et al., 2007)

### Identification of core genes based on statistical calculations

The correlation between module eigengenes and the gene expression of genes related to biotic stress was analyzed among the significantly correlated modules of interest associated with biotic stress in *Z. mays* silk. A heat map was used to represent the correlation values. The module membership (MM) is the association between each module eigengene and its gene expression as gene significance (GS), defined as the correlation between each trait and its gene expression (Tian et al., 2021). The MM and GS were determined to closely correlate the genes in a module with a cutoff value of MM >0.8 and a GS >|0.2| (Farhadian et al., 2021). The modules were correlated with the most significant DEGs common among relevant specific traits. All modules were considered core genes.

### Protein–protein interaction network establishment

A protein–protein interaction (PPI) network was constructed for the more significant common gene accession using the STRING database (v11) (Szklarczyk et al., 2019) by applying an interaction filter score >0.4 (medium confidence) and further visualized through Cytoscape (Doncheva et al., 2019). Within the networks, the parameters K-mean cluster score = 2, degree cutoff = 2, and maximum depth = 100 (Bandettini et al., 2012) were set. A subnetwork analysis was performed using molecular complex detection (MCODE) for the clustering connected. For the identification of key/essential genes from the PPI network, the CytoHubba (Chin et al., 2014) plugin of Cytoscape was used, which extracted the top 100 genes with the selected four scoring methods of CytoHubba, namely, maximal clique centrality (MCC), maximum neighborhood component (MNC), edge percolated component (EPC), and node–connect degree, respectively.

### Functional annotations and pathway analysis

To recognize and identify the functions of the significantly expressed genes, Biomart, ShinyGo, and UniProt (Kinsella et al., 2011; Consortium, 2015; Ge et al., 2020) were employed. The information was generated and further verified through BLASTx (Camacho et al., 2009) against PlantTFdb (Guo et al., 2007) and PRGdb version 3.0 (Osuna-Cruz et al., 2018) for the recognition of genes encoding for resistance, transcription factors, and specific pathways related to them. The DRAGO (PRGdb tool) pipeline also classified the R-gene classes and domain. In contrast, dbCAN2 with default parameters integrated with automated annotation tools of Diamond, HMMER, and Hotpep (Finn et al., 2011; Buchfink et al., 2015; Osuna-Cruz et al., 2018) enabled us to identify the polysaccharide degradation enzymes, also known as CAZymes.

## Results

To acquire a better understanding of the essential genes that are involved in the defense mechanisms in *Z. mays* silk against different fungal infections, a comparative study of the publicly accessible transcriptome information was carried out. The current study used DEG and WGCNA analyses to identify the core set of genes modulated in infected maize datasets. The core genes were further subjected to PPI for essential gene indication, followed by examining biotic stress determinants like TF, R-genes, and CAZymes. **Supplementary Figure S1A** provides a schematic of the analytical process. **Supplementary Figure S1B** displays the alignment data for each sample, and **Supplementary Figure S1C** illustrates the process by which readings were allocated to the genic feature count. The FastQC statistics showed that all the samples were of good quality, with above 80% of the samples lined up with the reference genome, and, further, that more than 70% of the reads were uniquely assigned to genic features.

### Evaluation of gene expression differences between fungal infection datasets

**Figure 1:**
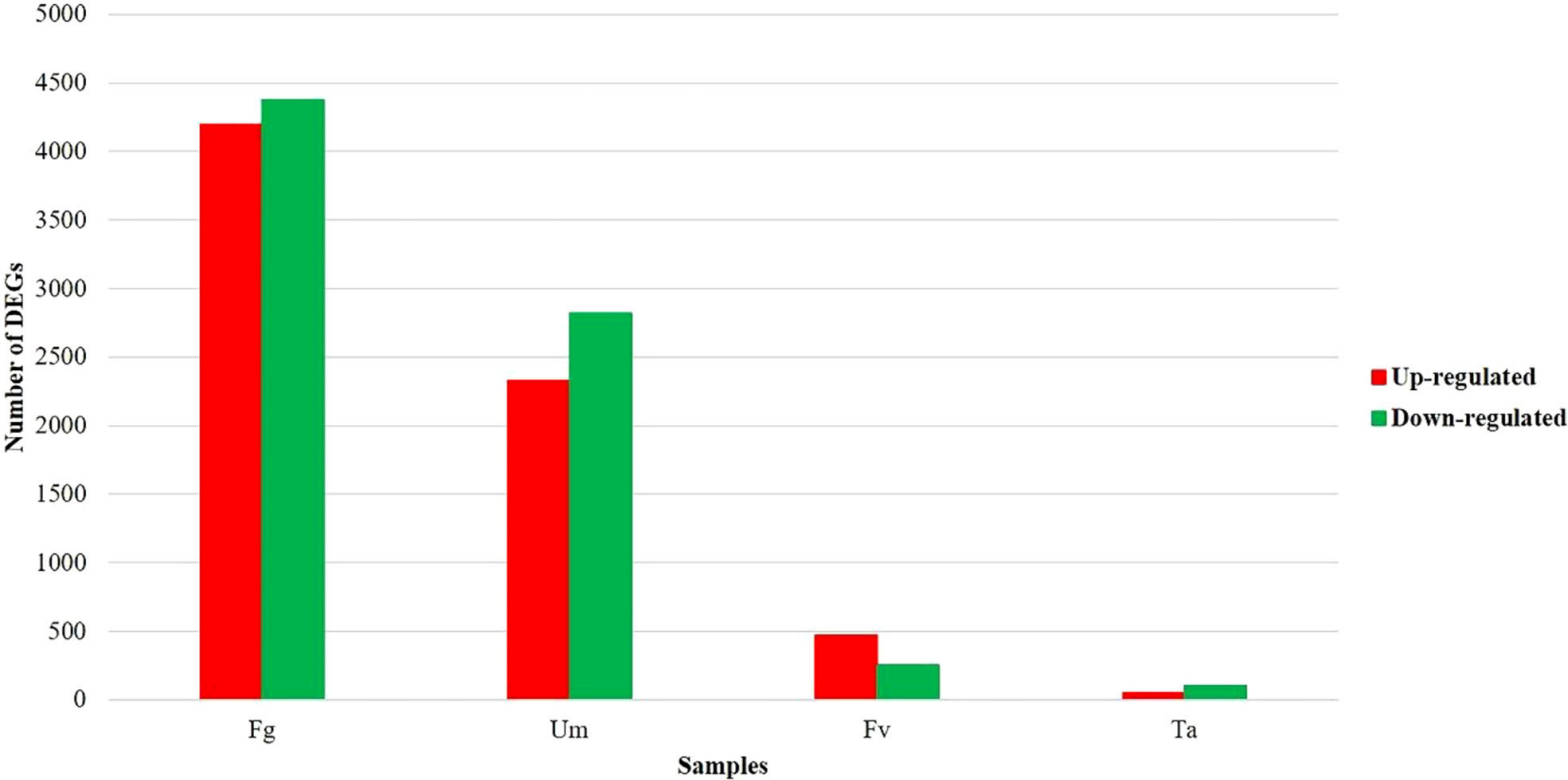
Distribution of differentially expressed genes involved in multiple fungal infections in silk of *Z. mays*. Fg, *F. graminearum*; Um, *U. maydis*; Fv, *F. verticillioides*; Ta, *T. atroviride*.

The two datasets were analyzed for differential expression using DESeq2, and genes with a *p*-value of 0.05 and |log_2_FC|>1.5 were determined to be significant DEGs. Approximately 33% of genes (14,694 and 14,808 of 44,303 genes) of datasets PRJEB13048 and PRJNA362306 (**Supplementary Table S2**) were differently expressed in pairwise differential expression analysis of fungus-infected *versus* control samples (**Table 1**) (**Supplementary Figure S1D1–4**). In the DEGs, approximately 28% (12,447) of genes appeared in both datasets (**Supplementary Figure S1E**). The number of DEGs varied in the fungal infections based on the comparison between up- and downregulated genes within controls. A comparative analysis of the DEG infections exhibited about 9,558 (22%) genes being expressed uniquely, among which roughly 5,043 (11%) genes were recognized to have been duplicated. **Figure 1** displays that 19.4% (8,577) of *Z. mays* silk genes were significantly expressed in Fg, 11.7% (5,146) were described in Um, 1.7% (723) were expressed in Fv, and 0.3% (155) were expressed in Ta infections, respectively (**Supplementary Table S3**). It is thus important to investigate which gene among them is responsible in regulating the expression under different fungal perturbations. The rationality variation in the value of the DEGs seemed to be caused by fungal infection (Yang et al., 2022), and this perhaps could be reasoned out with the differences observed in the sample sizes of the datasets, where the control of dataset A (Ca) had 7.5 GB, while the control of dataset B (Cb) had 2.5 GB. The concept well defends this examination that varied sample sizes and the spatiotemporal specificity of samples could influence the drastic variations in the different data sets (Wang, et al., 2021).

**Table 1:** Differentially expressed gene analysis of fungal infected transcriptomic data of maize silk.

Differentially expressed genes							
Ca vs. T				Cb vs. T			
Sample	Upregulated	Downregulated	Total	Sample	Upregulated	Downregulated	Total
Ca vs. Fg	5,017	5,667	10,684	Cb vs. Fg	6,230	6,371	12,601
Ca vs. Um	3,067	3,850	6,917	Cb vs. Um	5,060	5,136	10,196
Ca vs. Fv	2,392	2,628	5,020	Cb vs. Fv	1,210	1,028	2,238
Ca vs. Ta	2,410	2,934	5,344	Cb vs. Ta	287	256	543
Without duplicates			14,694	Without duplicates			14,808

Ca, control of dataset A; Cb, control of dataset B; T, treated; Fg, *F. graminearum*; Fv, *F. verticillioides*; Ta, *T. atroviride*; ; Um, *U. maydis*.

**Figure 2:**
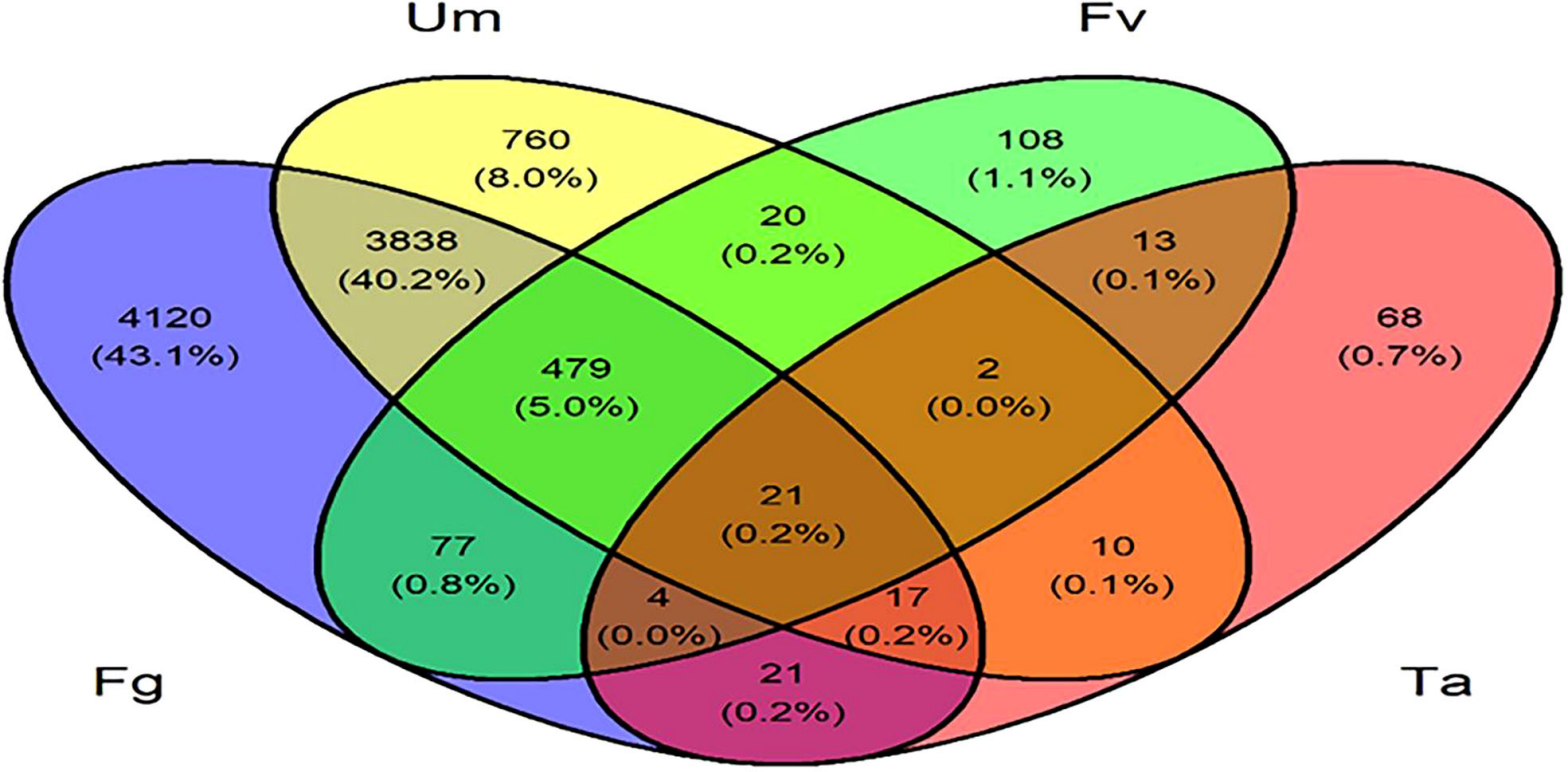
Venn diagrams of differentially expressed genes; the numbers in parentheses represent the total number of upregulated or downregulated genes in each combination of fungal infection in silk of *Z. mays*. Fg, *F. graminearum*; Fv, *F. verticillioides*; Ta, *T. atroviride*; Um, *U. maydis*.

Furthermore, a Venn diagram of total DEGs comparison between up- and downregulated genes within controls was examined in the interaction analysis of DEGs expressed in different fungal infections. In total, 21 (0.2%) genes were represented across the four different fungal infection settings. In the intersection of three fungal infection situations and specifically in Fg, Fv, and Ta infections, four genes (0.0%) were expressed. Additionally, in Fg, Ta, and Um infections, 17 genes (0.2%) were expressed. Fg, Fv, and Um infections expressed 479 (5.0%) genes. Two genes (0.0%) were expressed in Fv, Ta, and Um. The intersection between two fungal infection conditions, namely, Ta and Um, expressed 10 genes (0.1%). In Fv and Ta, 13 genes were expressed. Twenty genes (0.2%) were expressed under Fv and Um infection circumstances. Only Fg and Um expressed 3,838 (40.2%) genes. In the presence of both Fg and Fv infections, 77 (0.8%) genes were expressed. In Fg and Ta, 21 (0.2%) genes were expressed. Upon infection with a single fungal isolate of Fg, 4,120 (43%) genes were expressed, while 760 (8%) genes were expressed during the Um infection, and 108 (1.1%) and 68 (0.7%) genes were expressed in Fv and Ta infections, respectively (**Figure 2**).

These genes involved different physiological activities in *Z. mays*, including hormone response, secondary metabolism, phosphorylation, photosynthesis, cell wall organization, control, replication, and response to stimuli. Therefore, our basic premise is that these genes might have different gene expression patterns and counts depending on their natural genetic makeup. While some processes overlapped in samples of other fungal infections, Fg infection displayed higher gene expression levels in the *Z. mays* silk.

### Identification of the core genes using WGCNA and DEG analysis

**Figure 3:**
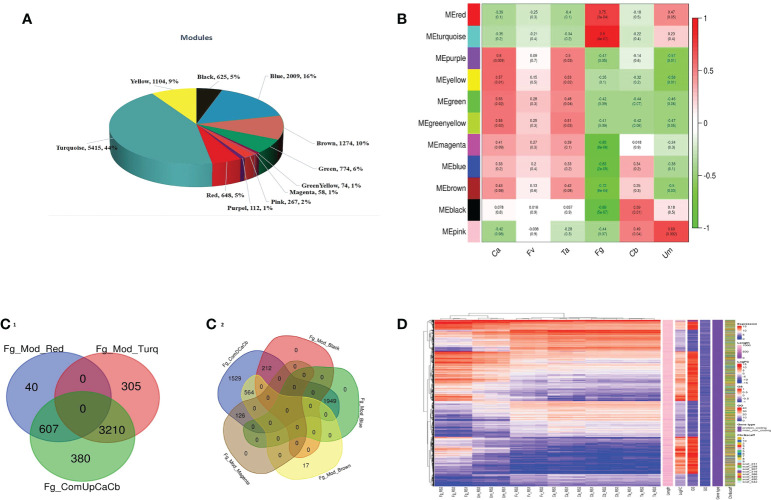
WGCN analysis of differentially expressed genes (DEGs) between control A and B of datasets. **(A)** Range of gene distributions per module. **(B)** Correlation coefficients between modules and samples. Red to green color depicted levels of positive and negative regulation of the genes, respectively. **(C)** The intersection between significant DEGs and module genes. Upregulated/positive correlation (C1); downregulated/negative correlation (C2). **(D)** Complex heat map of core genes (color codes have different classes).

Weighted gene co-expression network analysis of 12,447 common genes was carried out to identify the core genes involved in fungus defense. The WGCNA analysis resulted in 11 modules, with 58 genes in the small modules to 5,415 genes in the most significant modules (**Figure 3A**). A correlation of module eigengenes to disease trait data was performed, with a cutoff value of significance |GS| >0.5 and *P*-value <0.05. Out of 11 modules, purple, yellow, and green-yellow modules were associated with both Ca and Ta, the green module with Ca, red and turquoise modules with Fg, black with Cb, pink being positively correlated with Um, and magenta, blue, brown, and black modules being negatively correlated with Fg **(** **Figure 3B**). Interestingly, none of the modules was significantly associated with Fv. A further intra-modular analysis based on the gene significance (GS) and module membership (MM) of genes identified vital genes in the six modules for the Fg trait. A filter of |MM >0.8 and GS >|0.2| was applied for essential gene identifications. The up- and downregulated genes were compared to the genes in modules with relevant specific traits (Yang et al., 2020). We were able to infer that the up- and downregulated core genes in modules 3,817 (607 and 3,210, red and turquoise modules) and 2,851 (1,949, 564, 212, and 126; blue, brown, black, and magenta modules) were highly stable with DEGs in *F. graminearum* affected silk (**Figure3C**1-2). It can also be seen that the average (“logFC value of control a + control b/2) log_2_ fold change value of differently expressed genes was used to construct the complex heat map (**Figure 3D**). The complicated heatmap representation of 6,668 core genes, logFC (DEGs), and GS (WGCNA) exhibited approximately equal Fg fungal infection (**Supplementary Table S4**).

### Establishment of a protein–protein interaction network and identification of essential genes

**Figure 4:**
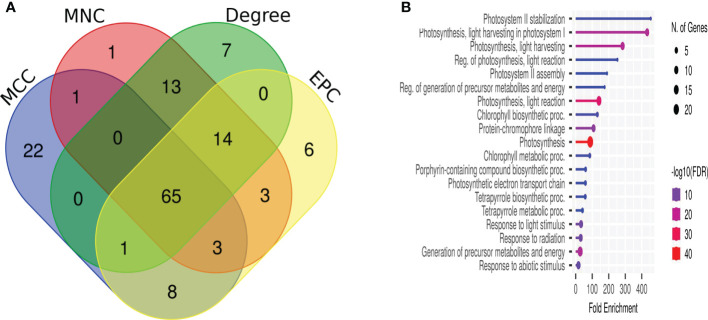
Protein–protein interaction network assembly of core genes. **(A)** Venn diagram of the top 100 essential genes based on the CytoHubba four scoring method. **(B)** The biological process enrichment of core genes of cluster 1.

We extracted the protein–protein interaction network with a medium confidence score >0.4 of *F. graminearum*-affected *Z. mays* silk 6,668 core genes that matched with 3,325 STRING genes (**Supplementary Table S5**). Network visualization and analysis were performed using Cytoscape, which identified 2,879 genes as nodes and 29,918 edges. No interaction was observed for the remaining 446 genes. Out of 86 clusters, only 12 MCODE scores were more significant than four of the MCODE clustering (**Tables 2**, **S6**). The PPI discovered 65 essential genes to be common in all four scoring techniques, such as MCC, MNC, EPC, and node–connect degree of CytoHubba (**Figure 4A**). Cluster 1 (35 nodes and 1,096 edges) contained downregulated genes, had the highest MCODE score (30.444), and overlapped the CytoHubba scoring. The enrichment analysis of the different biological processes of these essential genes was evaluated using the online enrichment tool ShinyGO. Substantial genes were enriched in photosynthesis, generation of precursor metabolites and energy, light reaction, and response to light stimulus (**Figure 4B**), indicating that a maximum number of genes were involved in the photosynthesis process.

**Table 2:** MCODE cluster of protein–protein interaction of core genes.

Cluster	Score	Nodes	Edges
1	30.444	35	1,096
2	16.324	36	604
3	10.696	45	492
4	8.383	46	394
5	7.15	39	286
6	6.682	43	294
7	5.31	28	154
8	5.25	15	84
9	5.176	16	88
10	5	7	40
11	4.571	13	64
12	4.214	27	118
Total		350	3,714

### Functional annotations of differentially expressed genes

A total of 14,601 DEGs were functionally identified in several fungus-affected samples. Overall, 773 high functional categories with 46.6, 45.7, 21.5, and 8.8% genes conforming to biological processes, molecular functions, cellular components, and pathways were identified, respectively (**Table 3**). In *F. graminearum* (Fg) infections to the *Z. mays* silk, the up-regulated genes gene ontology (GO) enrichment analysis revealed biological process categories with the following GO terms: protein phosphorylation (347 genes of the DEGs), defense response (133 genes of the DEGs), phosphorylation (423 genes of the DEGs), phosphate-containing compound metabolic process (543 genes of the DEGs), cell surface receptor signaling pathway (50 genes of the DEGs), phosphorus metabolic process (544 genes of the DEGs), and response to biotic stimulus (83 genes of the DEGs), response to external biotic stimulus (75 genes of the DEGs). Upregulated genes likewise contain 179 biological processes, 149 molecular functions, eight cellular components, and nine functional pathway categories (**Supplementary Table S7** **.FgU**). The downregulated genes GO enrichment analysis revealed biological process categories with the following GO terms: polysaccharide metabolic process (124 genes of the DEGs), carbohydrate metabolic process (261 genes of the DEGs), cellular glucan metabolic process (79 genes of the DEGs), glucan metabolic process (80 genes of the DEGs), cellular polysaccharide metabolic process (91 genes of the DEGs), polysaccharide biosynthetic process (72 genes of the DEGs), photosynthesis, light harvesting in photosystem I (16 genes of the DEGs), cellular carbohydrate metabolic process (108 genes of the DEGs), cell wall organization or biogenesis (104 genes of the DEGs), photosynthesis, light reaction (40 genes of the DEGs), likewise, in down-regulated genes contain 149 of biological processes, 49 of molecular functions, 53 of cellular components, and 15 pathway functional categories (**Supplementary Table S7** **.FgD**). In Um infections to the *Z. mays* silk, GO enrichment analysis of the DEGs identified biological process categories with the following GO terms: regulation of RNA biosynthetic process (242 genes of the DEGs), regulation of RNA metabolic process (245 genes of the DEGs), regulation of nucleobase-containing compound metabolic process (247 genes of the DEGs), defense response (47 genes of the DEGs), regulation of cellular macromolecule biosynthetic process, regulation of macromolecule biosynthetic process (250 genes of the DEGs), regulation of cellular biosynthetic process, regulation of biosynthetic process (251 genes of the DEGs), protein phosphorylation (181 genes of the DEGs), and nucleic acid-templated transcription (246 genes of the DEGs). In the upregulated genes,110 genes were involved in biological process, 103 genes performed molecular functions, eight genes were responsible for cellular component, and four genes are responsible for pathways (**Supplementary Table S7** **.UmU**), and in downregulated genes, 95 were detected for biological process, 38 genes for molecular functions, 56 and 19 for cellular components, and 19 pathways, respectively (**Supplementary Table S7** **.UmD**). In Fv infections of the *Z. mays* silk, GO enrichment analysis of the DEGs identified biological process categories with the following GO terms: defense response (28 genes of the DEGs), cell surface receptor signaling pathway (13 genes of the DEGs), response to biotic stimulus (17 genes of the DEGs), response to bacterium (11 genes of the DEGs), defense response to other organisms (15 genes of the DEGs), protein phosphorylation (48 genes of the DEGs), response to oxidative stress (17 genes of the DEGs), response to external biotic stimulus, defense response to fungi (nine genes of the DEGs), cell wall polysaccharide catabolic process, xylan catabolic process (four genes of the DEGs), photosynthesis, light-harvesting (four genes of the DEGs), cell wall macromolecule catabolic process (four genes of the DEGs), and phenol-containing compound biosynthetic process (four genes of the DEGs). Upregulated genes contain 126 biological process, 93 molecular process, six cellular components, and five pathways (**Supplementary Table S7** **.FvU**), downregulated gene contain 31 biological processes, nine molecular functions, and 14 cellular components (**Supplementary Table S7** **.FvD**). GO terms were significantly enriched in Ta infections caused in *Z. mays* silk. The following GO terms were identified: response to the stimulus (six genes of the DEGs), response to stress (five genes of the DEGs), catabolic process (four genes of the DEGs), response to endogenous stimulus (three genes of the DEGs), regulation of metabolic process and regulation of cellular process (three genes of the DEGs), cellular response to stimulus (three genes of the DEGs), cell wall organization or biogenesis (two genes of the DEGs), regulation of cellular process (18 genes of the DEGs), regulation of metabolic process (16 genes of the DEGs), developmental process (seven genes of the DEGs), response to stimulus (five genes of the DEGs), cellular component organization (three genes of the DEGs), cell cycle process (three genes of the DEGs), and cellular component organization or biogenesis (two genes of the DEGs). Upregulated genes likewise contain 32 biological process, 21 molecular functions, and 14 genes for cellular components as identified (**Supplementary Table S7** **.TaU**), and the downregulated genes contain 33 biological processes, 21 molecular functions, and 17 cellular components with high-level GO categories (**Supplementary Table S7** **.TaD**).

**Table 3:** Gene set enrichment analysis of DEGs.

Sample	Expression	DEGs	Biological process		Molecular function		Cellular component		Pathways	
			Gene	High-level GO category	Gene	High level GO category	Gene	High-level GO category	Gene	High-level GO category
Fg	Upregulated	4,197	1,838	197	2,291	149	526	8	341	9
	Downregulated	4,380	1,730	149	1,235	49	1054	53	349	15
Um	Upregulated	2,325	1,013	110	2,270	103	326	8	122	4
	Downregulated	2,821	1,876	95	542	38	1,063	56	371	19
Fv	Upregulated	468	178	126	239	93	90	6	92	5
	Downregulated	255	22	31	39	9	53	14	0	0
Ta	Upregulated	50	107	32	18	21	10	14	0	0
	Downregulated	105	31	33	29	21	15	17	0	0

DEGs, differential expression genes; GO, gene ontology; Fg, *F. graminearum*; Fv, *F. verticillioides*; Ta, *T. atroviride*; Um, *U. maydis*.

### Functional annotations of core genes

**Figure 5:**
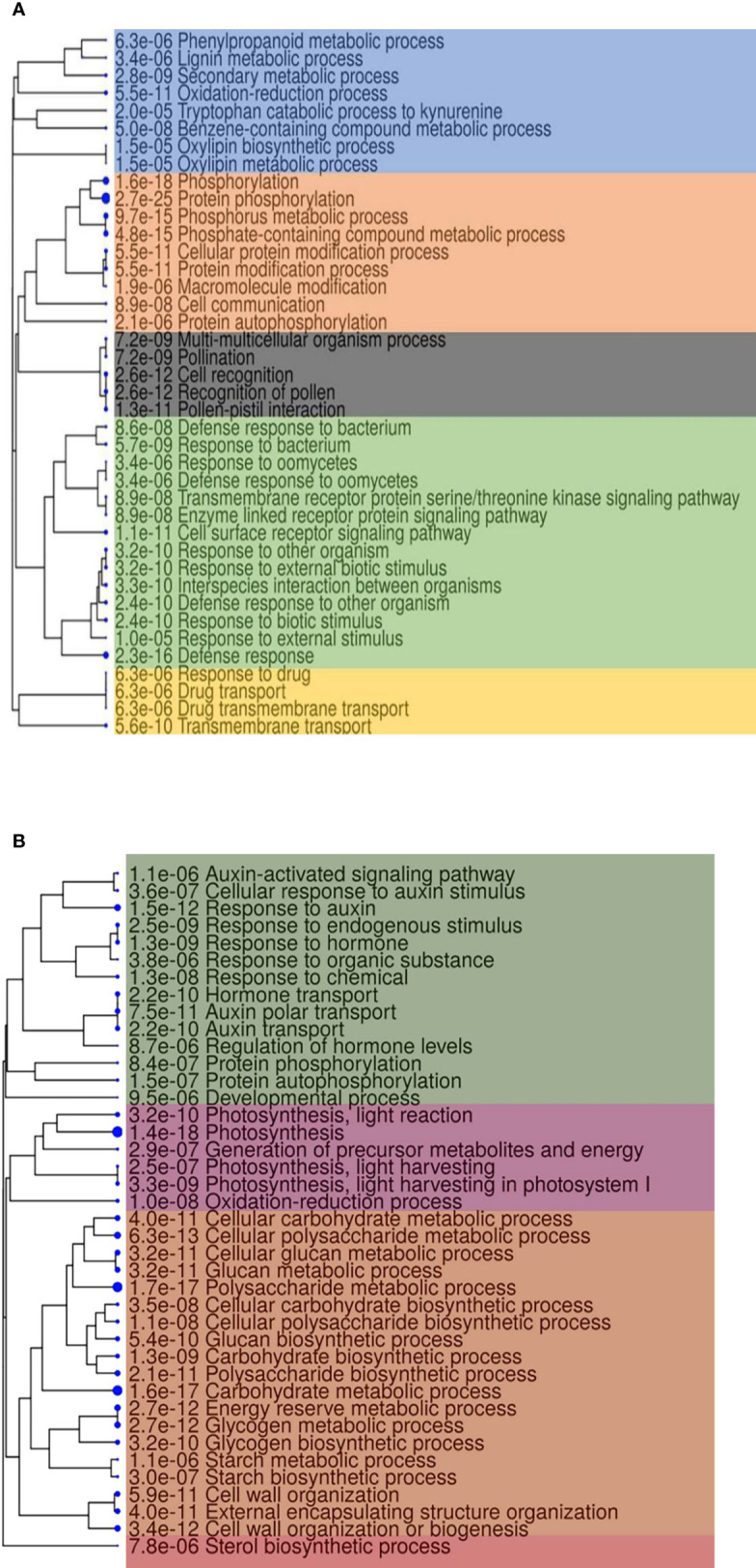
A hierarchical clustering tree summarizing the correlation among the top 40 Gene Ontology biological process enrichment of core genes. **(A)** Upregulated and **(B)** downregulated. Biological processes with numerous shared genes are clustered together. Blue dots with numbers indicate the false discovery rate values. High significance is designated with bigger dots.

The functional enrichment analysis of core genes with a false discovery rate <0.05 showed 704 higher-level GO categories in which 3,082, 3,254, 1,360, and 582 genes were involved in biological processes, molecular functions, cellular components, and pathways (**Supplementary Table S8**). Core genes with highly enriched GO terms positively included biological processes to stimulus–response, defense responses against fungus, and phosphorylation (**Figure 5A**). The GO annotations of downregulated genes showed that they belong to different biological processes (**Figure 5B**) in Fg infection of *Z. mays* silk.

### Identification of transcription factors, CAZYme genes, and resistance genes in core genes

**Figure 6:**
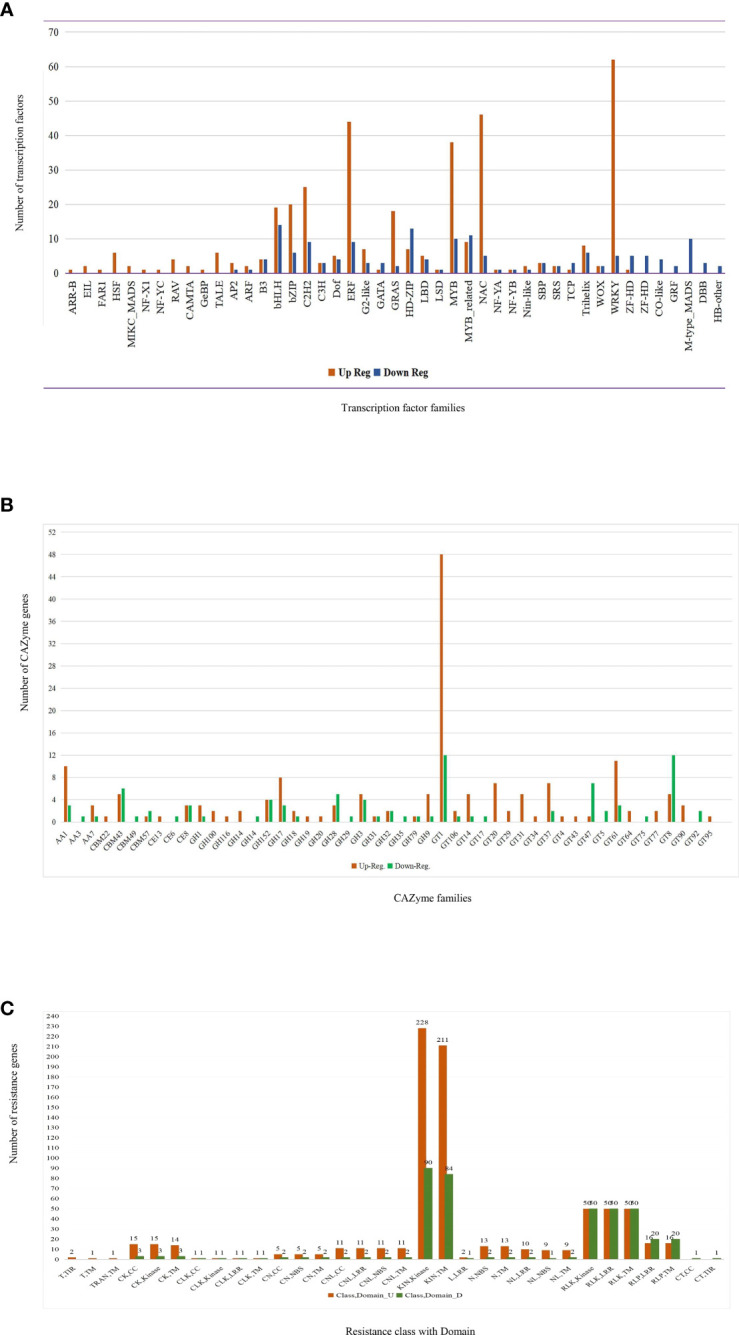
Circulation of differentially expressed genes. **(A)** Transcription factor, **(B)** CAZyme annotation, and **(C)** resistance genes identified from core genes. (GT, Glycosyl Transferase; CBM, Carbohydrate-Binding Modules; GH, Glycoside Hydrolase; AA, Auxillary Activities; CE, Carbohydrate Esterases; KIN, Kinases; RLK, Receptor-like kinases; RLP, Receptor-like proteins consisting an LRR repeat; CK, coiled-coil structure; CLK, Contains coiled-coil; LRR and Kinase domains CNL, CC-NB-LRR; L, Leucine-Rich Repeat; N, Nucleotide-binding site; T, Toll-Interleukin Receptor-like; TRAN, transmembrane helix).

Among the 45 transcription factor (TF) classes, WRKY, NAC, ethylene-responsive factor (ERF), MYB, C_2_H_2_, basic helix–loop–helix (bHLH), and GRAS TFs were highly differentially expressed in infected silk. The heat shock transcription factor, transcription activator-like effectors, RAV, M-type-MADS, and ZF-HD showed up- and downregulation **(** **Figure 6A**). Out of 520 TFs from core genes, 410 TFs matched with ShinyGo functional annotation of DEGs. Three TFs, namely, bHLH-0, DRE-binding protein3/ERF, and MYB-110, were upregulated in all the infected samples. AP2-EREBP-115, C2C2-Dof-26, and Homeobox-59 were upregulated in Fg, Ta, and Um infections. Five WRKY, four NAC, and MYB, three bHLH, two AP2-EREBP, G2-like, and one bZIP TF family were upregulated, and two TFs, namely, bHLH-161 and Homeobox-60/71, were downregulated in Fg, Fv, and Um infections. In total, 228 TF genes were expressed in Fg and Um infections, while 147 TF genes were expressed in a Fg infection (**Supplementary Table S9**).

Core genes contained 257 genes for carbohydrate-active enzymes (CAZYme), while dbCAN2’s diamond, hmmer, and hotpep databases equally shared 169 and 88 up- and downregulated genes. Specific expressions of 16 and 10 modules were found in up- and downregulated genes, respectively, while 24 modules were found in both (**Figure 6B**). These genes belong to different modules, namely, glycosyl transferase (GT), glycoside hydrolase (GH), carbohydrate-binding modules (CBM), auxillary activities (AA), and carbohydrate esterases (CE). A further comparison with DEGs from other fungi (Fv, Ta, and Um) was conducted. The 20 CAZyme-related genes were detected in three fungus-infected silk samples (Fg, Fv, and Um), 123 genes in Fg and Um infections, and two genes in Fg and Fv infections; 112 CAZyme-related genes were only expressed in Fg infections of *Z. mays* silk (**Supplementary Table S10**).

Upon screening of resistance (R) genes from significant core genes, 346 and 174 up- and downregulated R-genes (**Supplementary Table S11**) were found. These 520 R-genes are divided into 13 classes and seven domains. The majority of kinase and transmembrane (TM) domains comprised of the KIN class (**Figure 6C**), and the other classes were receptor-like kinases (RLK), receptor-like protein (RLP), receptor-like proteins consisting of an LRR repeat (RLP), contains coiled-coil and kinase (CK), nucleotide-binding site (N), CC-NBS-LRR (CNL), NL (NBS-LRRs), *etc*. Compared with Fv-, Ta-, and Um-infected silk, the putative DUF26-domain receptor-like protein kinase family protein showed a positive expression in all fungal infections. In the Fg, Fv, and Um conditions, 48 R-genes were considerably expressed. In the Fg and Um infections, 222 R-genes were expressed, with three R-genes in Fg and Fv infections and 227 R-genes expressed in Fg condition. The receptor-like serine/threonine-protein kinase, putative leucine-rich repeat receptor-like protein kinase family protein, and protein kinase superfamily were all present in more significant amounts in Fg and Um than in Fv and Ta conditions. These expression patterns played a crucial role during signal transduction and other biological functions.

## Discussion

Fungi are the second major biotic factor that reduce crop yield after insects. Some of the major fungal diseases of maize are *Gibberella* ear rot, *Fusarium* ear rot, corn smut, brown spot, *etc*., which cause considerable yield losses up to 42% (Thompson and Raizada, 2018). In addition, fungi produce many mycotoxins, leading to poisoning and quality deterioration (Agostini et al., 2019). However, there is a lack of research information regarding the direct comparative studies with respect to multiple fungal infections (Fg, Fv, Ta, and Um) in maize silk and identification of abundant genes in these four fungal infections. The current study is based on computational approaches of the publicly available transcriptome data of *Z. mays* silk infected with multiple fungi focused on core genes identification by differential expression analysis followed by co-expression analysis through WGCNA. We identified 14,694 and 14,808 DEGs of control datasets of A and B (**Supplementary Table S2**). In further simplification, 4,519 and 5,125 genes were determined by comparing the up- and downregulated genes within controls. The up- and downregulated genes were compared to genes in the modules of WGCNA with relevant specific traits, and core genes were identified as described in the method and represented in **Figure 3C**. Our comparative study found that, in Fg infection conditions, more genes were affected compared to other Fv, Ta, and Um fungal infections. Twenty-one (21) most prominent genes identified in this study were expressed in all four fungal infections of maize silk. Many significant genes were identified, which were common to conditions caused by three and two fungi. Moreover, 4,120, 108, 68, and 760 genes were uniquely expressed in Fg-, Fv-, Ta-, and Um-affected silk (**Figure 2**).

### In samples affected with four fungi

**Figure 7:**
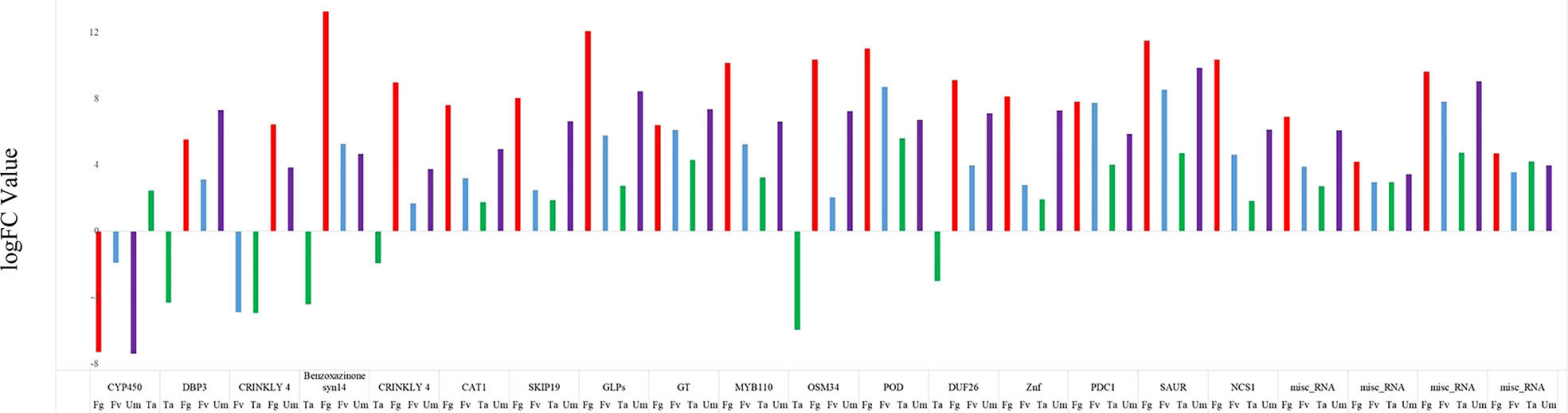
Illustration of differentially expressed genes of common genes of four fungal infections. *F. graminearum* (Fg), *U. maydis* (Um), *F. verticillioides* (Fv), and *T. atroviride* (Ta) are designated as red, purple, blue, and green, respectively.

Twenty-one (21) DEGs were identified in all four infections **(** **Figure 2** **)** which showed different expression values and functions in maize silk. The analysis of four fungus-affected samples revealed that these genes showed a higher significance in Fg infection than in other fungal infections. The upregulated expression of 21 genes was found except for the downregulation of CYP 450 in Fg, Fv, and Um and BP3, CRINKLY 4, OSM 34, DUF 26, and benzoxazinone in Ta along with CRINKLY 4 in Fv infections (**Figure 7**). According to Li and co-workers, the peroxidase (POD) enzyme controls the lengthening of germ tubes to shield maize kernels from fungal diseases (Li et al., 2018). It is a fact that POD genes were found to be upregulated in all samples of maize silks infected by fungi, with logFC values of 11.02 in Fg infections, 8.7 in Fv infections, and 5.59 and 6.7 in Ta and Um infections, respectively. These are suggestive that the POD genes that we detected in our study also have a similar function. Interestingly, Um had a higher expression of the DBP3 protein gene than the other fungal infections in maize silks. Reports suggest that multiple steps downstream of the ABA-independent route show its significance in the regulation of abiotic stress (Joshi et al., 2016). Conversely, our research indicates that DREB protein synthesis promotes a defensive activity against fungal infections, especially Um. The expression of benzoxazinone is highest in the Fg and Fv tissues, followed by Um, and lowest in Ta infections. The current study coincides with the impact of another report which suggests that it has an effect on pests and antifungal activity (Cantillo et al., 2017). A DEG analysis revealed that, in Ta, the cytochrome p450 (CPY450) gene is upregulated despite showing downregulation in other fungal infections. This gene plays a variety of roles in plant defense, including the biosynthesis and catabolism of phytohormones and other secondary compounds (Xu et al., 2015; Lambarey et al., 2020; Li and Wei, 2020; Pandian et al., 2020). In addition, the expression pattern of CRINKLY4, a kinase family protein, showed a variable expression in fungus-affected silk. These proteins influence the shape of the cell size and the epidermal development in maize leaf (Becraft et al., 1996). CAT1 expression increases during microbial infections and hinders plant growth, according to a previous study (Yang et al., 2014). Our study demonstrated that fungal pathogen assaults on maize silks activated CAT1-related genes. The results also suggest that it could change the metabolic activity during fungal invasion in maize silks (Vina-Vilaseca et al., 2011; Yang et al., 2014). It is reported that SKIP19 protein genes influence and respond to biotic and abiotic stress and play a key role in soybean pollen tube germination and salt and drought tolerance (Chen et al., 2008; Yang et al., 2008; Chang et al., 2009; Ren et al., 2020). There is variable expression in Fg and Um infections, but the strong expression in Fv and Ta infections confirms the SKIP19 gene’s role in *Z. mays* Fv and Ta defense. The osmotic-like protein (OSM34) in plants, animals, and fungi improves host defense and immune defense against biotic and abiotic stress (de Jesús-Pires et al., 2020). Our research found that OSM34 protein genes were upregulated in Fg, Fv, and Um infections but downregulated in Ta infections in accordance with the roles mentioned. DUF26, which is upregulated in all fungal infections under study, except Ta, belongs to the receptor-like protein kinase sub-family; its domain plays a crucial role in stress resistance and antifungal defense (Liu et al., 2021). Putative RING zinc finger domain superfamily proteins have ubiquitin–protein ligase activity and help plant growth and development in *A. thaliana* (Gao et al., 2015; Kim et al., 2019). Small auxin-up RNA is a member of the auxin-responsive gene family that is upregulated in all the fungal infections considered in the present inquiry with logFC 11.5 in Fg, 8.5 in Fv, 4.7 in Ta, and 9.8 in Um. Previous research using microarray data profiling identified these genes as highly expressed in the roots and leaves but less in seeds, which is essential in plant growth and development (Chen et al., 2014; Zhang et al., 2021). S-norcoclaurine synthase proteins exhibit a defense response and have a signaling receptor activity, and four miscellaneous RNA genes were identified.

### In samples affected by three fungi

Within the Fg, Fv, and Um fungal infection conditions, 479 expressed genes out of 502 are intersectionally connected. These genes were relatively high in defense responses, photosynthesis, detoxification, and secondary metabolic processes. In any case, the Fg conditions have a higher expression value than those observed with Fv and Um infections (**Supplementary Table S12**). Seventeen genes are highly expressed in Fg, Ta, and Um samples; these genes are involved in the DNA-binding transcription factor activity and are upregulated in these Fg and Um samples but downregulated in Ta infections (**Supplementary Table S12**). Several genes implicated in the light reaction of photosynthesis were discovered to be highly expressed during abiotic stress conditions (McNinch et al., 2020). This finding clearly indicates that the presence of fungal pathogen in the *Z. mays* silk may be a crucial factor controlling the photosynthesis processes, functions of the plant cell surface receptor signaling pathway, and hydrogen peroxide catabolic process. In our study, the terpene synthase genes TPS6 (Ensembl id: Zm00001eb412960) and TPS11 (Ensembl id: Zm00001eb412980) were significantly upregulated in Fg, Fv, and Um infections caused in maize silk (**Supplementary Table S12**). A group of researchers (Huffaker et al., 2011) observed that terpene synthase (TPS6 and TPS11) proteins involve the plant pathogen’s defense. TPS6 and TPS11 are transcribed only in the leaves and roots of *Z. mays*. TPS6/TPS11 function in terms of resistance to Um infections and tumor formations (van der Linde et al., 2011) and have a role in the production of several antibiotics (Huffaker et al., 2011).

### In two fungus-affected silk

During Fg and Um infections, a total of 3,838 were intersectionally connected. Seventy-seven genes (77) were found to be commonly expressed in Fg and Fv infections, while 20, 21, and 13 genes were common between Fg and Ta, Fv and Um, and Fv and Ta infections, respectively (**Supplementary Table S13**). These genes were highly expressed in response to stimulus and stress. As suggested by Thompson and Raizada (2018), the maize silk have a defense mechanism against fungal infections, with wounds being the most susceptible to damage caused by Fg. Apart from maize silk, studies conducted by Reid et al. (1992) and du Toit and Pataky (1999) also identified that Fg and Um almost take the same time period for a successful infection in ear heads. Yang et al. (2018) suggested that E3 ligase under drought tolerance of *Z. mays* plays a crucial role in enabling plants to effectively and efficiently cope with environmental stress. In our results, RING-type E3 ubiquitin transferase was positively expressed under Fg- and Um-infected silk of *Z. mays* (**Supplementary Table S13**). Photosynthesis, chlorophyll a-b binding protein, and light reaction photosynthesis I and II reaction center genes were highly downregulated. HVA22-like protein was downregulated, wherein HVA22 specifically inhibits GA-induced PCD/vacuolation of aleurone cells in barley (Guo and Ho, 2008). Glucanendo-1,3-beta-glucosidases (β-1,3-glucanases) protein genes have negative regulation, and these proteins play a significant role against the fungal pathogen by degradation of the cell wall. Lozovaya et al. (1998) found a positive correlation between the *Aspergillus flavus* fungus-infected kernel of maize silk and β-1,3-glucanases. Gao et al. (2017) found that GDSL esterase/lipase participates in immunity through lipid homeostasis in rice. In Fg and Um infection conditions, 10 GDSL genes were downregulated, and three upregulated genes were found. Huo et al. (2020) reported that 10 genes strongly contribute to male fertility, such as immature tassels, meiotic tassels, and others.

### Maize silk affected by individual fungus

Maize silk infected with Fg activated more genes and was involved in phosphorylation (**Supplementary Table S14**). This suggests that phosphorylation may be one of the initial events in a putative signal transduction pathway leading to the post-translational modification of a protein that controls cell cycle, development, growth, and stress responses. The research group of Palmer et al. (1993) reported that blue light induces phosphorylation in *Z. mays* plant mediated by an enzyme which belongs to the Ser/Thr class of kinases (Luan, 2002). Furthermore, a large number of genes were found to be involved in carbohydrate metabolic process when affected with the Fg pathogen, with the carbohydrate metabolism genes being downregulated during the process. When maize silk was infected with the Um pathogen, most genes responded to stimulus, stress, oxidation–reduction (redox) reactions, and biological processes like cell cycle (de la Torre et al., 2020). Genes involved in the cell cycle process were downregulated. It has been found that the cell cycle regulation and appressorium morphologenesis are delicately linked. The given primary function of the appressorium is to aid in the invasion of the plant tissue and the subsequent proliferation inside the host (de la Torre et al., 2020). Significant expression patterns were not observed with Fv and Ta infections. In summary, the Fg infections cause more damaging effects compared to other fungal infections. The analysis revealed that 4,355 were interconnected during intersectional studies **(** **Figure 2** **)** with Fg and Um pathogen conditions belonging to *Nectriaceae* (Fg) and *Ustilaginaceae* (Um).

During biotic stress, it was observed that genes associated with photosynthesis were downregulated as reported by researchers (Doke et al., 1996; Zhu and Li, 2015), which was in agreement with our results. In Fg infections, variations were observed with cell wall-related genes (**Supplementary Table S14**), as fungal pathogens are known to secrete pectinases, xylanases, cellulases, and ligninases (Sharma, 2016) which can cause plant cell wall degradation during the infection process.

### Protein–protein interaction network establishment in the infected silk of *Zea Mays*

We identified 3,325 proteins from the string databases of core genes. The network was simplified into 12 highly sub-connected clusters (**Table 2**) and identified essential proteins in the network based on the CytoHubba scoring method. The PPI network revealed that cluster 1 has 35 downregulated core proteins that infected the maize silk plant and were involved in biological processes (**Figure 4B**), such as photosynthesis. A research team (Horst et al., 2008) proposed that Um infection to *Z. mays* leaves reduced the photosynthetic rate and maintained the nutrients as well as influenced the chlorophyll content on a time scale (Kshirsagar et al., 2001). Wang et al. (2021) proposed the light harvesting in photosystem 1, a biosynthetic/metabolic process positively expressed in *Gibberella* stalk rot disease in *Z. mays* plant. Moreover, in other clusters 2 and 3, UMP pyrophosphorylase protein is involved in UMP biosynthesis *via* salvage and L-tryptophan biosynthesis. It has an intermediate role in benzoxazinoid biosynthesis with indole-3-glycerol phosphate in the chloroplast (Richter et al., 2021). Photorespiration, the pathway used to regenerate 2-phosphoglycolate metabolism, plays an essential role in photosynthesis in higher plants and is localized in chloroplasts (Eisenhut et al., 2008; Bräutigam and Gowik, 2016). Trehalose-6-phosphate synthase protein has a role in sugar-induced signaling pathway, and its function has different stages in the plant on growth and development. Iordachescu and Imai (2008) found that plant trehalose levels are typically low. They can change in response to shoot drought, salt, and cold stress challenges in roots and shoots. Another group of researchers (Henry et al., 2015) also reported that trehalose pathway genes were highly affected under saline conditions. Furthermore, these genes were downregulated with the involvement of fructose-bisphosphate aldolase (FBA) protein in various pathways, namely, glycolysis, carbohydrate degradation, and other physiological and biochemical processes. These biological processes include plant defense, response to biotic stress, plant growth, plant development, regulation of secondary metabolites, signal transduction, and Calvin cycle (Lv et al., 2017) and have been documented in other plant species including *Z. Mays*, *A. thaliana*, and *Oryza sativa* (Mininno et al., 2012) under abiotic stress conditions like salt, drought, heat and cold conditions. Our study finds FBA protein in cluster 2 and is downregulated in Fg infections in maize silk but upregulated in wheat to improve the enzyme activity and CO_2_ concentrations in green plant tissues during development (Lv et al., 2017). In cluster 4, upregulated genes involved phosphotransferase, which has a vital role in the hexose metabolism pathway, which is part of carbohydrate metabolism, to generate glucose-6-phosphate for glycolysis. GRMZM2G076075_P02, glucose-6-phosphate isomerase, is also involved in the glyconeogenesis process, whereas GRMZM2G161245_P01; malate dehydrogenase, is an enzyme that participates in the citric acid cycle from the conversion of malate into oxaloacetate (using NAD+) and also has a *vice versa* reaction (Takahashi-Íñiguez et al., 2016). In *Pisum sativum*, a 280% increase in malate dehydrogenase enzyme activity was observed with respect to *Fusarium* wilt diseases in comparison to control pea plants (Reddy and Stahmann, 1975) and downregulated proteins GRMZM2G074158_P01 and GRMZM2G085577_P01; α-1,4-glucan phosphorylase belongs to the glucosyltransferase family, and these enzymes have an important role in starch and metabolism pathway given the reversible transfer of glucosyl units from glucose-1-phosphate to the non-reducing end of α-1,4-d-glucan chains with the release of phosphate (Rathore et al., 2009). Cluster 6 has 45 nodes with 295 edges and one hub node, which is a cover scoring method. The expression of the protein GRMZM2G137151_P01 (1-deoxy-D-xylulose 5-phosphate synthase, DXS) genes was mainly in *Artemisia annua* leaf and flowering buds (Zhang et al., 2018a). In our study, the expression of this protein is upregulated in Fg fungal infections in the maize silks. A similar result was reported by Cordoba et al. (2011) who demonstrated plastid localization in *Z. mays* leaves. DXS catalyzes the first reaction that converts pyruvate and glyceraldehyde-3-phosphate to 1-deoxy-D-xylulose 5-phosphate in the methylerythritol phosphate pathway (Tambasco-Studart et al., 2005; Zhang et al., 2020). The remaining cluster 5 and nodes, namely, 7, 10, 11, and12 (**Table 2**), do not cover up the pathways under the high scoring method within the top 100 (**Supplementary Table S15**).

### TF, CAZymes, and R-genes in maize silks

In our current endeavors, while analyzing the transcriptome data from different fungal infections and infections caused in corn silks, several TFs, R-gene, and CAZymes have been identified from core genes that serve as a molecular switch to interact with cis-acting transcription factor binding sites and directly control the transcriptional regulation of plant genes (Kimotho et al., 2019; Soni et al., 2020). In our study, 45 families were identified from 367 and 153 up- and downregulated transcripts of plant genes respectively (**Figure 6A**), such as WRKY, NAC, AP2/ERF, MYB, C2H2, bHLH, bZIP, *etc*. (**Supplementary Table S9**). One of the significant plant-specific transcription factors is encoded by the WRKY gene family, discovered in several plant species (Ma et al., 2021) which have highly expressed genes. A team of researchers from the Louisiana State University (Fountain et al., 2015) reported similar results in maize with respect to the resistance and susceptibility to *A. flavus* fungal infection. WRKY plays a vital role in biotic stress and is involved in PAMP signaling and multiple defense responses through mitogen-activated protein kinase (MAPK) signaling, especially in sensing pathogen effectors or PAMP, and also interacts with resistance (R) protein (Meng and Zhang, 2013). Numerous studies have shown that a significant proportion of WRKY TFs are involved in disease response *via* the jasmonic acid (JA) signaling pathway (Ma et al., 2021). These TFs act as repressors or activators of basal defense responses (Windram et al., 2012). Similarly, NAC TFs participate in gene transcription regulations (Journot-Catalino et al., 2006), development, and stress response (Farhadian et al., 2021) and are the second highly expressed TFs in endosperm and kernels than in roots and stems that were known to regulate starch synthesis (Olsen et al., 2005; Xiao et al., 2021). Our study observed that NAC TFs were highly expressed in Fg infection of silk of *Z. mays* compared to other fungal infections such as Fv, Ta, and Um. Many plants have seen ERF TFs involved in disease resistance with phytohormone-mediated fungal defense (Luo et al., 2019), such as ERF activity in JA-mediated defense responses (Grennan, 2008; Jin et al., 2017). In *A. thaliana*, DREB TFs represent a large part of the AP2/ERF superfamily (Agarwal et al., 2017; Hrmova and Hussain, 2021). An MYB transcription factor is a more prominent family involved in multiple biological functions in the plants, such as primary and secondary metabolite reactions, regulating the plant growth and development, cell morphogenesis, and response to biotic and abiotic stress (Cao et al., 2020; Duan et al., 2021). However, in plants, MYB TF works as an activator for transcription that triggers G2/M-specific gene expression (2011; Haga et al., 2007). Our findings indicated that MYB-related genes might be involved in the *Z. mays* pathogen response since the expression profile of the MYB-related gene family in *Z. mays* and soybeans exhibits a wide range of variation with time following a Um infection (Du et al., 2013). Researchers studied the expression of bHLH TFs in the young leaf, root, and auricular tissue of Z*. mays* which have a high expression while being involved in plant development (Murre et al., 1994; Zhang et al., 2018b). After about a decade, the bHLH TF was identified in a study conducted on *A. thaliana* that acts as the target of JAZ protein and negatively regulates JA-mediated plant defense and development (Song et al., 2013). Wei et al. (2012) reported that 125 bZIP genes were found, which encode 170 proteins in *Z. mays* tissue; 18 bZIPTF were significantly upregulated as expressed in silk. These TFs regulate different biological processes such as floral development, seed formation, response to biotic and abiotic stress (Katagiri et al., 1989), starch synthesis in rice endosperm and maize kernel, and they saw that starch synthesis genes have a similar expression pattern (Wang et al., 2013).

The invasion of fungal pathogens through penetration to the silk of *Z. mays* enhanced the defense mechanism against pathogens through activation of the gene encoding cell wall-associated proteins, of which UGTs (UDP-glycosyltransferases) have been found in *Z. mays* and other species in investigations (Duan et al., 2021). It is involved in the production of phytohormones, metabolites, growth, development, and biotic and abiotic stress (Li et al., 2014; Rehman et al., 2018). UDP members of the GT1 family catalyze, help the biosynthesis of oligo- and polysaccharides, and transfer sugar residues from nucleotide donor substrates to receptor substrates or a developing carbohydrate chain (Hoffmeister et al., 2001; Jayaprakash et al., 2021). GT1-related genes were substantially up-regulated in the Fg-affected silk of *Z. mays*. Cao et al. (2008) have reported that the GT1 family is the most prominent family in all three species. Aside from the GT1 family, rice’s top five GT families include the GT2, GT4, GT8, GT31, and GT47 families in *A. thaliana* and *Populus* species (poplar). GT8 and GT47 classes were highly downregulated in Fg fungal infections (Kong et al., 2019), and GT8 family was found to play an essential role in plant cell wall formation which is considered critical for growth and development. The cold and saline conditions significantly caused upregulation to aid in salt stress tolerance. Cao et al. (2008) distinguish that most GT47 genes have a low expression in the different development stages of rice. Similarly, the second most highly expressed CAZyme family was GH (**Supplementary Table S10**), which is involved in the carbohydrate metabolic process (GO:0005975) to cleave glycosidic bonds in various forms of glucan, glycosides, and glycoconjugates. These also have industrial use and biotechnological applications to develop bio-fuel (xylanases, cellulases, *etc*.) and are useful in pharmaceutical research (Roy et al., 2020). This study also identified CAZymes like CBM, CEs, and AA enzyme to be involved in lignin catabolic process with less gene expression in differential expression analysis **(** **Figure 6B** **)**.

We were also successful in identifying 13 classes of R-genes which consist of seven different domains from core genes. In this particular analysis, the KIN class was found to be a major class of R-genes consisting of kinase (KIN) and kinase transmembrane KIN-TM domains **(** **Figure 6C**). Similarly other major R-gene classes identified were receptor-like kinases (RLK), receptor-like protein (RLP), and receptor-like-protein consisting of a leucine-rich repeat (LRR), which play a crucial role in plant development as well as response to biotic and abiotic stress (De Hoff et al., 2009). Resistance genes are also known as adult plant resistance genes or quantitative resistance genes. R-genes have been identified within host and pathogen cells (Jones et al., 2014). The defense response in plants depends on the pathogen’s attack, such as phytohormones involved in defense responses and salicylic acid which controls the biotrophic pathogens (Kelley et al., 2012). Jasmonic acid (JA)-dependent and ethylene (ET)-dependent signaling pathways regulate the necrotrophic pathogens (Bari and Jones, 2009; Birkenbihl and Somssich, 2011). Most plant disease R proteins have been seen to contain a series of LRRs, a nucleotide-binding site (NBS), and a putative amino-terminal signaling domain. Thus, NBS–LRR protein activations are a phenomenon that changes the structure as well as nucleotide-binding status (DeYoung and Innes, 2006). DUF26 is a subfamily receptor-like protein kinase. It has 90% identity with the cysteine-rich receptor (CRR)-like protein kinase; its domain has antifungal activity and an essential role in stress resistance (Liu et al., 2021). It has been discovered in *Arabidopsis* plant that the CRR RLKs (CRKs) include two DUF26, CRK9, CRK26, and four DUF6 domains. These domains are involved in ABA signaling *via* regulating the ABA responses to seed germination, development, abiotic stress, and potential antifungal agents (Quezada et al., 2019). DUF26 domain protein genes were positively expressed in our study in all infected fungal conditions. Two proteins of DUF26 domain—AFP1 and AFP2—were found in Um-infected *Z. mays* apoplastic fluid (Ma et al., 2018) which are upregulated and act as an antifungal, thus increasing the resistance to fungal pathogens. In total, 48 R-genes are significantly expressed in Fg, Fv, and Um conditions, 222 R-genes have been expressed in Fg and Um conditions, three R-genes were found in Fg and Fv infections, and 227 R-genes are described in Fg condition. Subsequently, R-genes such as protein kinase superfamily proteins, receptor-like serine/threonine-protein kinase (EC 2.7.11.1), and putative leucine-rich repeat receptor-like protein kinase family protein were more significantly expressed in Fg and Um compared to other fungal infections (Fv and Ta). Alam et al. (2010) reported that LRR–RLK gene expression was significantly low in a salt-stress condition compared to other abiotic stresses. Additionally, putative receptor-like protein kinase, leucine-rich repeat protein kinase family protein, disease resistance RPP13-like protein 4, L-type lectin-domain containing receptor kinase IX.1, and LRR family proteins were significantly expressed only in Fg and Um (**Supplementary Table S11**). It is known that the LRR sequence participates in a strong PPI.

## Conclusion

A crucial clue to biotic variables affecting the *Z. mays* plant was discovered after the available silk of *Z. mays* transcriptome information was unified on NCBI and re-examined. Based on a comparative study using statistical estimates such as the DEG, the expression level of genes and the behavior of similar coding proteins have been found very distinct during multiple fungal infections in the silk of *Z. mays*. The 21 DEGs have been found in all four fungus-infected silk of *Z. mays*. The up- and downregulated genes were compared to the genes in the modules of WGCNA with relevant specific traits and identified core genes. In the current study, 520 from TFs, 169 from CAZyme, and 520 from R-genes among 6,668 core genes are involved in Fg infection in *Z. mays* silk, but not in the other fungal systems examined (Fv, Ta, and Um) for their transcriptomic datasets of *Z. mays* silk. The present study supports that the immunity stimulus and resistance genes are upregulated, while the downregulated genes are involved in photosynthesis, cell wall organization, pectin metabolism process, and response to auxin in the silk of *Z. mays*. We know that Fg and Fv belong to *Nectriaceae* family, but gene expression is very different, with 723 in Fv and 8577 in Fg. It is reported that Fg has an antagonistic relationship with Um, but the current study supports the probable similar function of the expressed genes during a fungal infection. Based on the transcriptome data analysis, we found that Ta does not affect the infected maize silk.

## Data availability statement

Publicly available datasets were analyzed in this study. The names of the repository/repositories and accession number(s) can be found in the article/supplementary material.

## Author contributions

AK, KRK, and PTVL designed the research. AK and KRK performed the analysis and analysed the results. AK wrote the initial manuscript. PTVL, RSD, and AA majorly reviewed and analysed the draft and finalized the manuscript. All authors contributed to the article and approved the submitted version.

## Funding

The fund for this study was received from Pondicherry University.

## Acknowledgments

The authors are thankful to the Department of Bioinformatics, Pondicherry University for providing all the necessary infrastructure for this work. AK is grateful to Pondicherry University for providing a Non-National Eligibility Test fellowship.

## Conflict of interest

The authors declare that the research was conducted in the absence of any commercial or financial relationships that could be construed as a potential conflict of interest.

## Publisher’s note

All claims expressed in this article are solely those of the authors and do not necessarily represent those of their affiliated organizations, or those of the publisher, the editors and the reviewers. Any product that may be evaluated in this article, or claim that may be made by its manufacturer, is not guaranteed or endorsed by the publisher.
