# Supplementary material for: Comparative transcriptome profiling and weighted gene co-expression network analysis to identify core genes in maize (Zea mays L.) silks infected by multiple fungi
Source: Front Plant Sci. 2022 Oct 27;13:985396. doi: 10.3389/fpls.2022.985396 (PMC9647128; doi:10.3389/fpls.2022.985396)
Supplement: Supplementary file 1 [file DataSheet_1.zip › Supplementary Files/SUPPLEMENTARY FIGURE S1.docx]

**Comparative Transcriptome Profiling and Weighted Gene Co-expression Network Analysis to Identify Core Genes in Maize (*Zea mays* L.) Silks Infected by Multiple Fungi**

Amrendra Kumar^1^, Kanak Raj Kanak^2^, Annamalai Arunachalam^3^, Regina Sharmila Dass^2^, PTV Lakshmi^1^*

^1^Phytomatics Lab, Department of Bioinformatics, School of Life Sciences, Pondicherry University, R. V. Nagar Kalapet, Pondicherry, India - 605014

^2^Fungal Genetics and Mycotoxicology Laboratory, Department of Microbiology, School of Life Sciences, Pondicherry University, Puducherry, India - 605014

^3^Postgraduate and Research Department of Botany, Arignar Anna Government Arts College, Villupuram, Tamil Nadu, India

*Corresponding author: [lakanna@bicpu.edu.in](mailto:lakanna@bicpu.edu.in), [lakshmiptv@yahoo.co.in](mailto:lakshmiptv@yahoo.co.in)


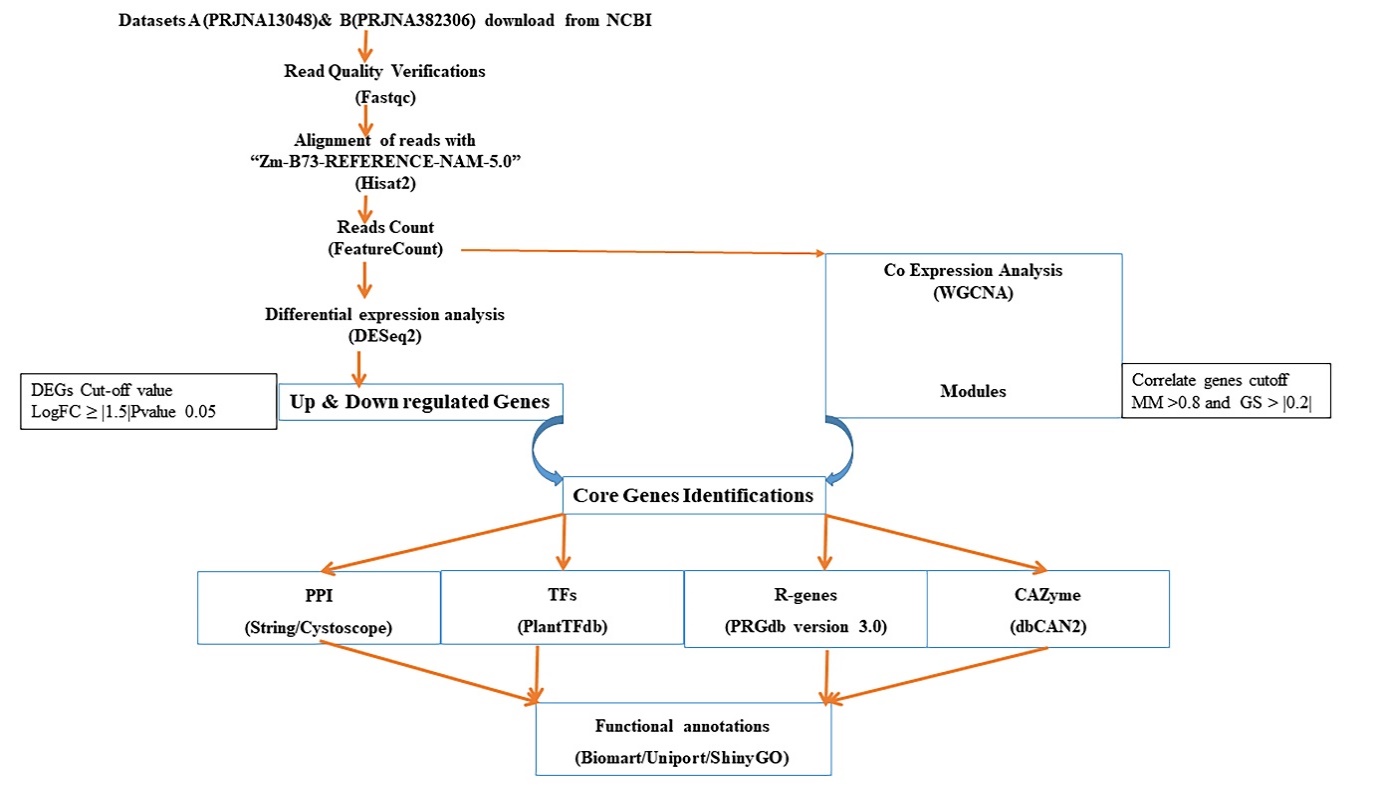
**Work-Flow**

**SUPPLEMENTARY FIGURE S1A |** Flowchart illustrating the study design (DEGs, differentially expressed genes; WGCNA, weighted gene co-expression network analysis; MM, Modules membership; GS, gene significance; PPI, protein-protein interaction; TF, Transcription factors; R, Resistance gene). Tools name is in small brackets.


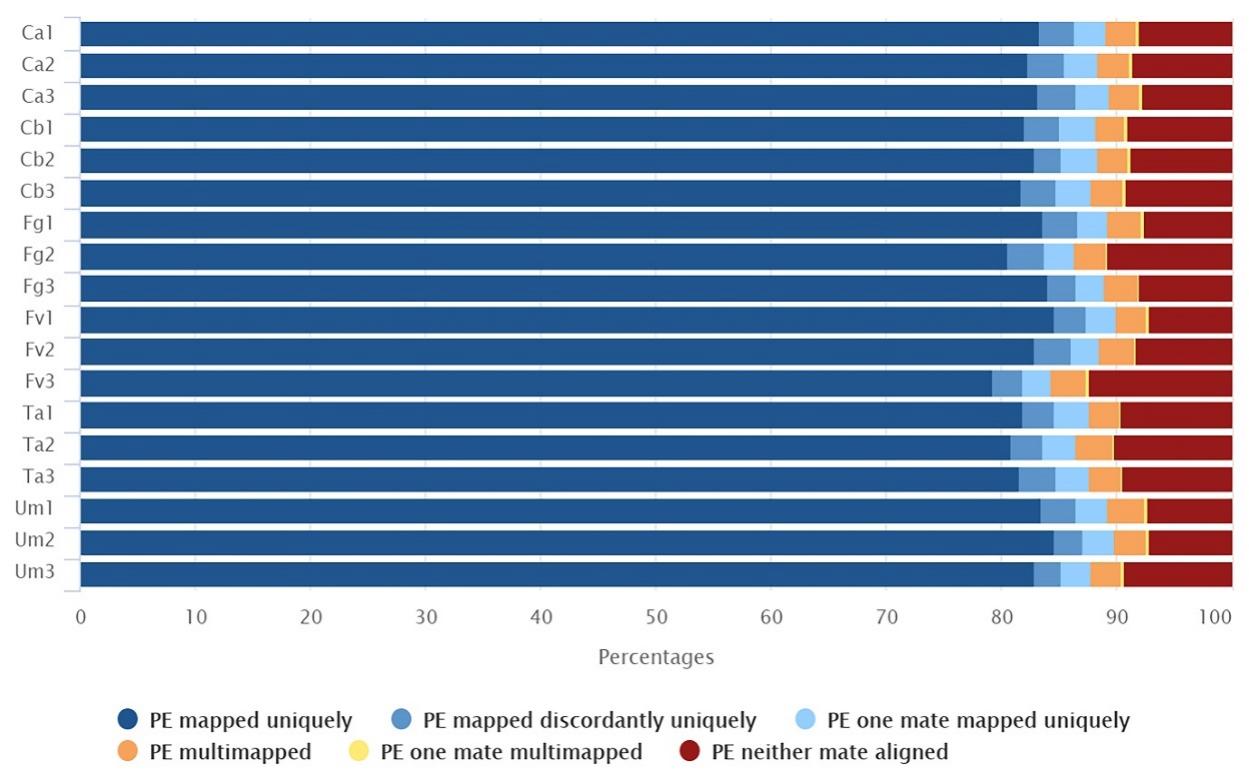


Replicates samples

**SUPPLEMENTARY FIGURE S1B |** Hisat2 pair end alignment score between read sequence and reference sequences (B73) of Z. mays silk


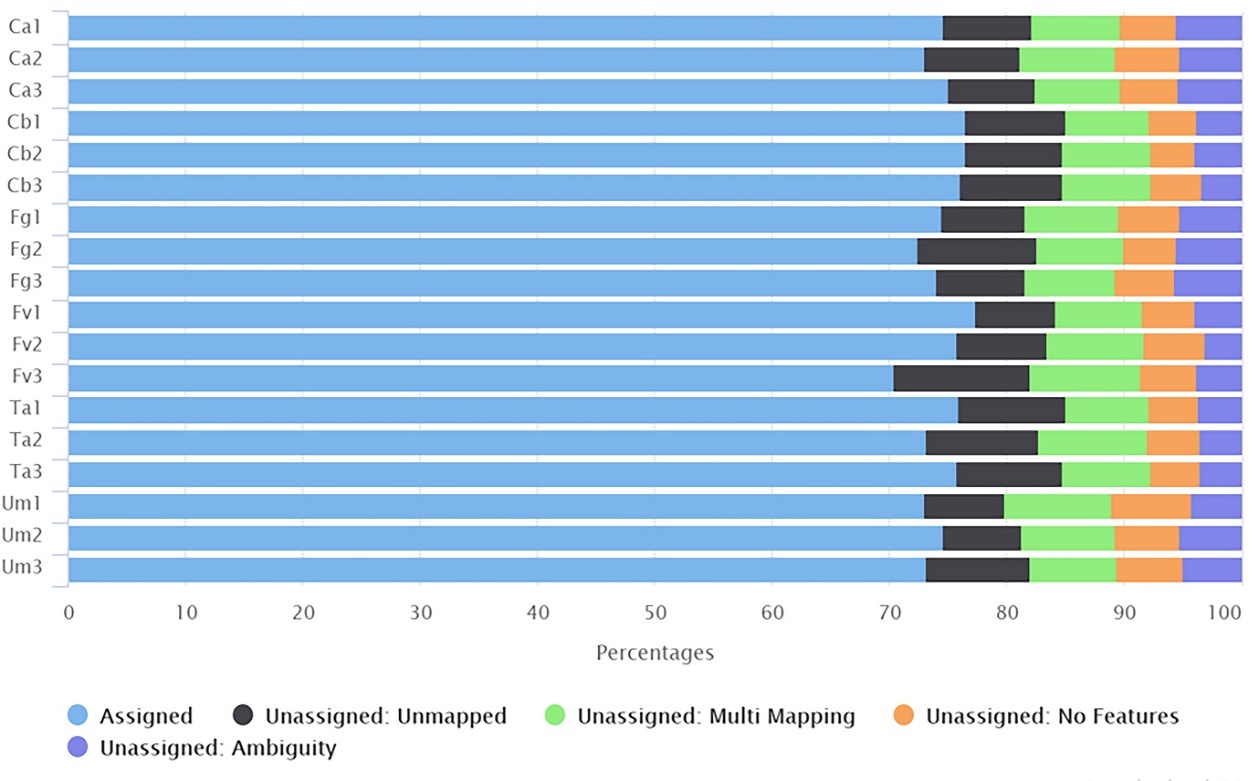


Replicates samples

**SUPPLEMENTARY FIGURE S1C |** Feature count assignment of alignment read sequences of samples


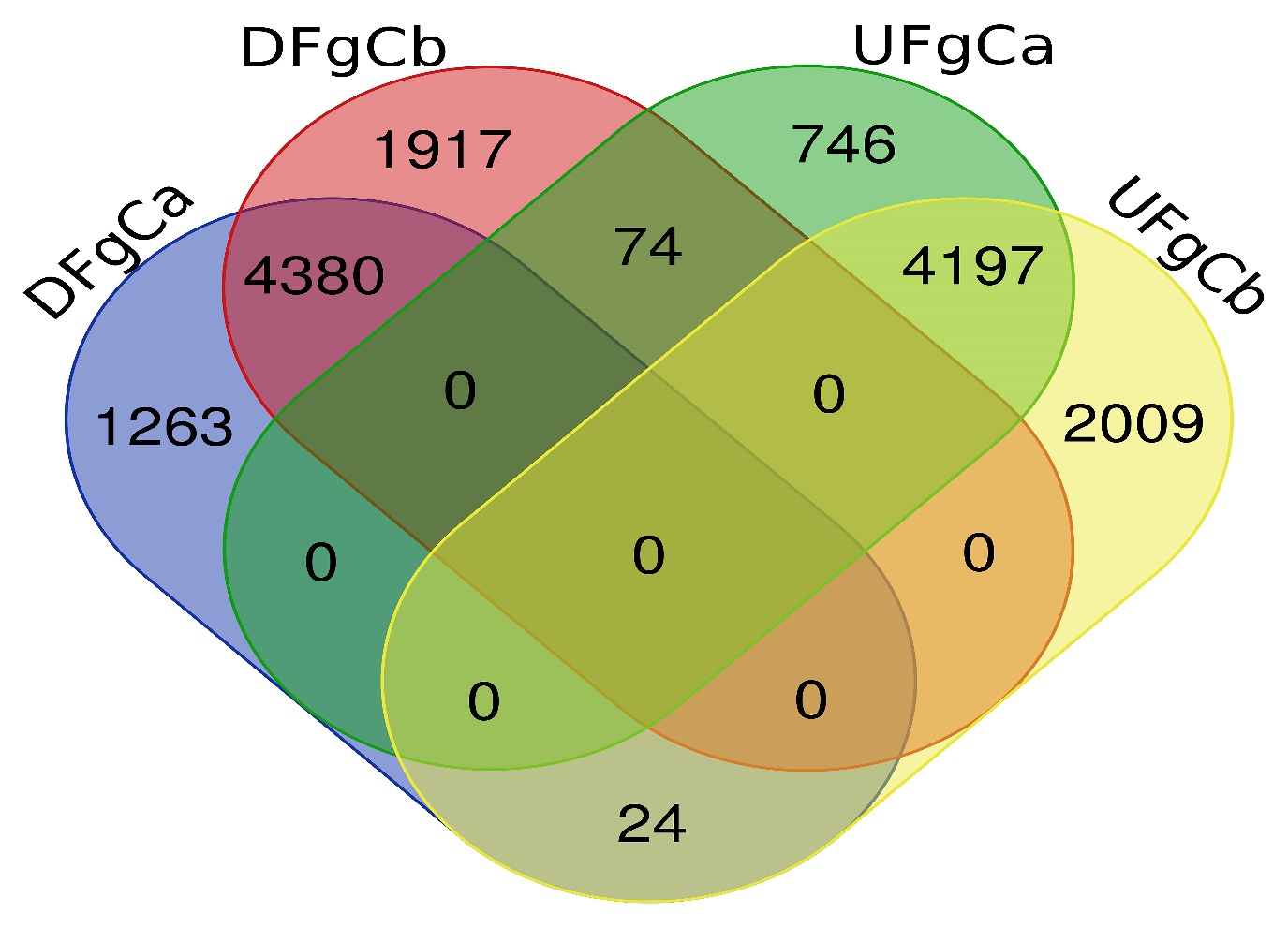


**(1)**


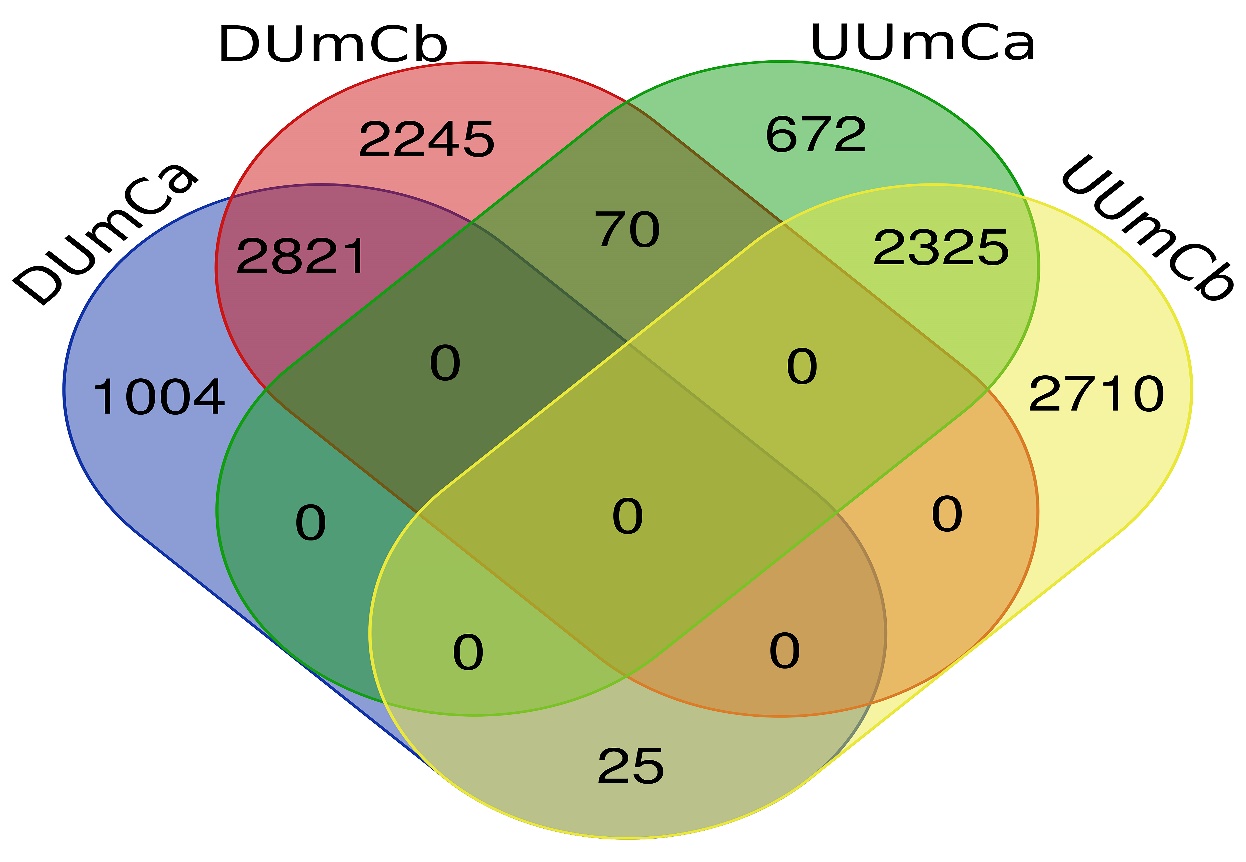

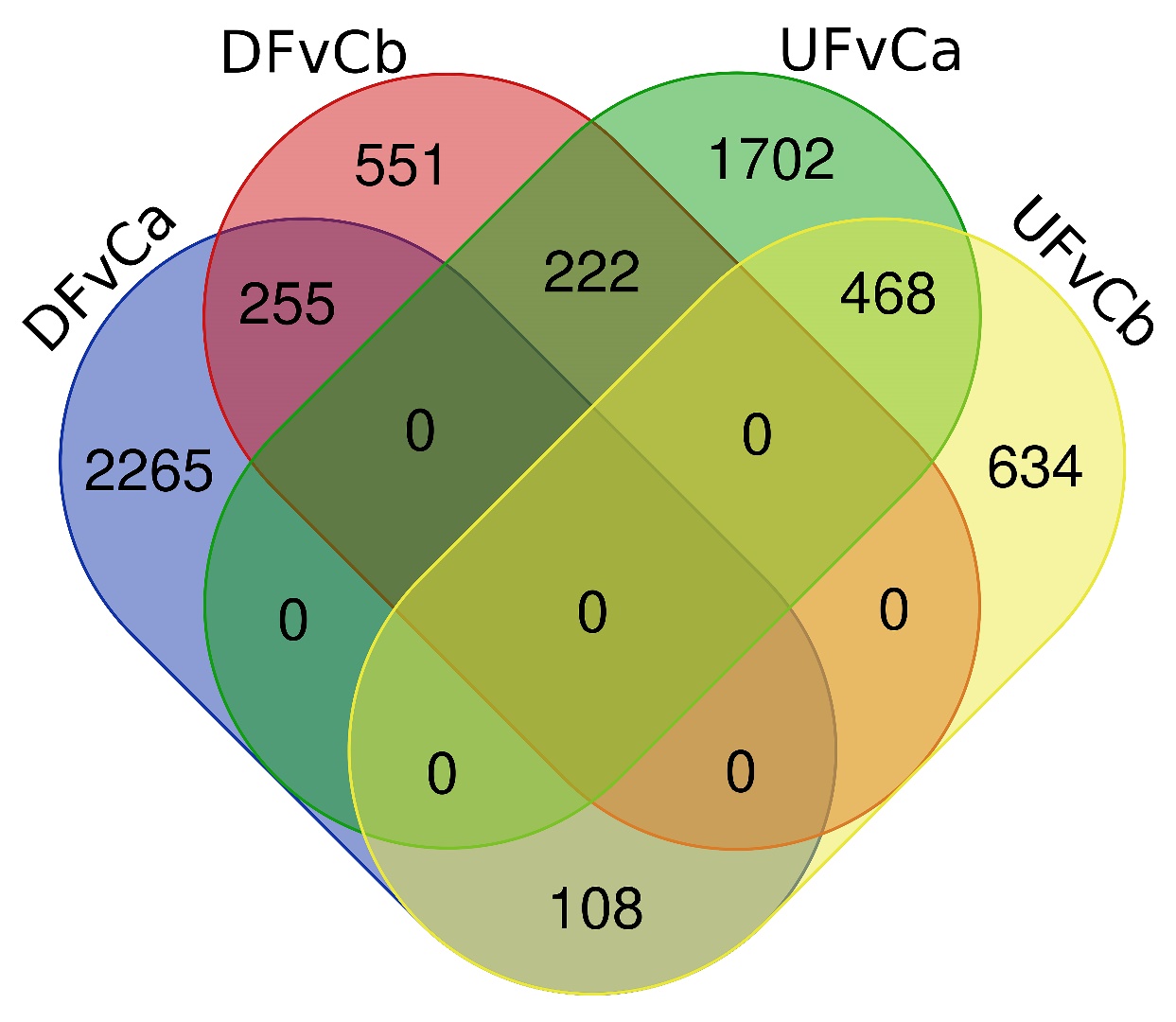


**(3)**

**(2)**


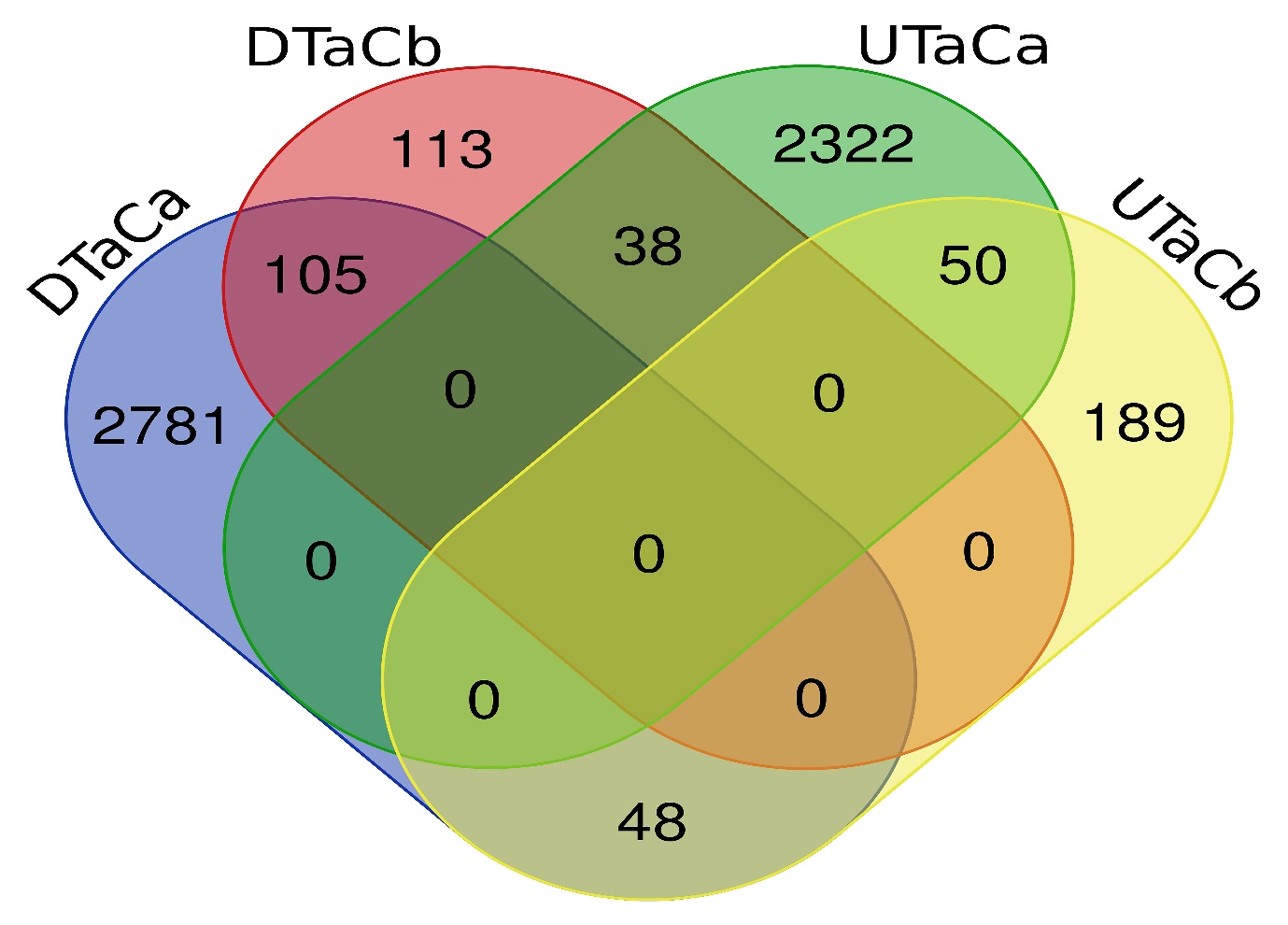


**(4)**

**SUPPLEMENTARY FIGURE S1D1-4** | Venn Digram based on the comparison between up- and down-regulated genes within controls. (1) Fg: *F. graminearum*; (2) Um: *U. maydis*; (3) Fv: *F. verticillioides;* and (4) Ta: *T. atroviride*; D: down-regulated; U: up-regulated; Ca: control of dataset A; and Cb: control of dataset B.


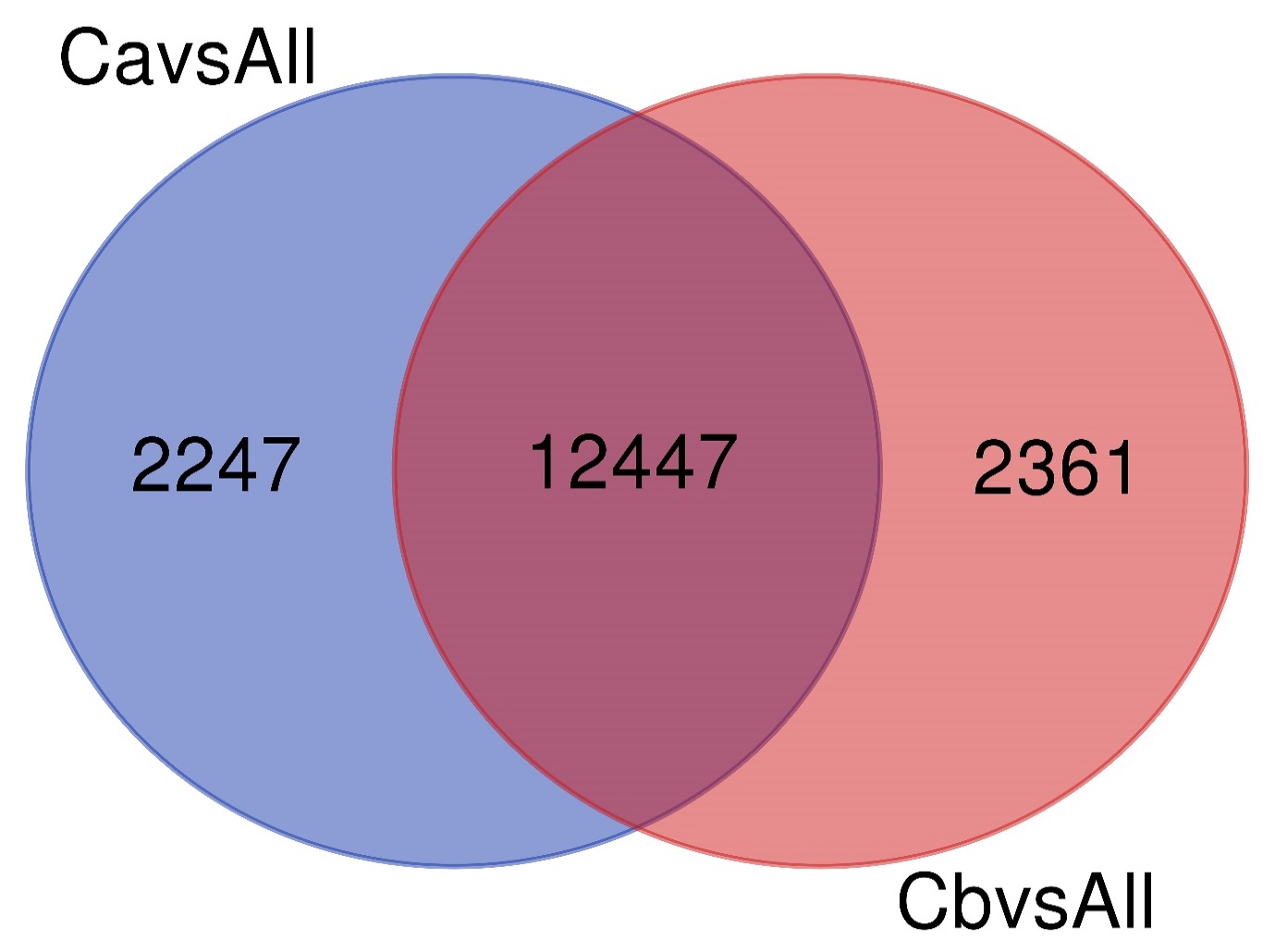


**Ca vs All**

**Cb vs All**

**SUPPLEMENTARY FIGURE S1E |** Common Genes identifications from the control A and B of datasets; Ca: Control of the dataset A; Cb Control of dataset B; All: *F. graminearum, U. maydis, F. verticillioides, T. atroviride* affected condition.
