# Supplementary material for: Comparative transcriptome profiling and weighted gene co-expression network analysis to identify core genes in maize (Zea mays L.) silks infected by multiple fungi
Source: Front Plant Sci. 2022 Oct 27;13:985396. doi: 10.3389/fpls.2022.985396 (PMC9647128; doi:10.3389/fpls.2022.985396)
Supplement: Supplementary file 1 [file DataSheet_1.zip › Supplementary Files/SUPPLEMENTARY TABLE S1.docx]

**Comparative Transcriptome Profiling and Weighted Gene Co-expression Network Analysis to Identify Core Genes in Maize (*Zea mays* L.) Silks Infected by Multiple Fungi**

Amrendra Kumar^1^, Kanak Raj Kanak^2^, Annamalai Arunachalam^3^, Regina Sharmila Dass^2^, PTV Lakshmi^1^*

^1^Phytomatics Lab, Department of Bioinformatics, School of Life Sciences, Pondicherry University, R. V. Nagar Kalapet, Pondicherry, India - 605014

^2^Fungal Genetics and Mycotoxicology Laboratory, Department of Microbiology, School of Life Sciences, Pondicherry University, Puducherry, India - 605014

^3^Postgraduate and Research Department of Botany, Arignar Anna Government Arts College, Villupuram, Tamil Nadu, India

*Corresponding author: [lakanna@bicpu.edu.in](mailto:lakanna@bicpu.edu.in), [lakshmiptv@yahoo.co.in](mailto:lakshmiptv@yahoo.co.in)

**Supplementary Table S1A|** Data information

| **Dataset A (PRJEB13048)** | |
| --- | --- |
| **Inoculation Infections** | **Run id of samples** |
| Normal Silk (Ca) | ERR1314961  ERR1314962  ERR1314963 |
| *Fusarium graminearum* (Fg) | ERR1314944  ERR1314945  ERR1314946 |
| *Ustilago maydis* (Um) | ERR1314965  ERR1314966  ERR1314967 |
| **Dataset B (PRJNA362306)** | |
| Normal Silk (Cb) | SRR5183834  SRR5183835  SRR5183836 |
| *Fusarium verticillioides* (Fv) | SRR5183837  SRR5183838  SRR5183839 |
| *Trichoderma atroviride* (Ta) | SRR5183840  SRR5183841  SRR5183842 |

**Supplementary Table S1B** |Experimental design of Differential Expression Gene (DEG) analysis

| **Control a from Dataset A vs Treated** | **Control b from Dataset B vs Treated** |
| --- | --- |
| Ca vs Fg | Cb vs Fg |
| Ca vs Fv | Cb vs Fv |
| Ca vs Ta | Cb vs Ta |
| Ca vs Um | Cb vs Um |
